# Activity-dependent compartmentalization of dendritic mitochondria morphology through local regulation of fusion-fission balance in neurons in vivo

**DOI:** 10.1038/s41467-024-46463-w

**Published:** 2024-03-08

**Authors:** Daniel M. Virga, Stevie Hamilton, Bertha Osei, Abigail Morgan, Parker Kneis, Emiliano Zamponi, Natalie J. Park, Victoria L. Hewitt, David Zhang, Kevin C. Gonzalez, Fiona M. Russell, D. Grahame Hardie, Julien Prudent, Erik Bloss, Attila Losonczy, Franck Polleux, Tommy L. Lewis

**Affiliations:** 1 Department of Neuroscience, Columbia University, New York, NY USA; 2 Mortimer B. Zuckerman Mind Brain Behavior Institute, Columbia University, New York, NY USA; 3 Aging & Metabolism Program, Oklahoma Medical Research Foundation, Oklahoma City, OK USA; 4 Neuroscience, Biochemistry & Molecular Biology, Oklahoma University Health Science Campus, Oklahoma City, OK USA; 5 Division of Cell Signalling & Immunology, School of Life Sciences, University of Dundee, Dundee, DD1 5EH Scotland UK; 6 Medical Research Council Mitochondrial Biology Unit, University of Cambridge, Hills Road, CB2 0XY Cambridge, UK; 7 The Jackson Laboratory, 600 Main Street, Bar Harbor, ME 04609 USA

**Keywords:** Cellular neuroscience, Mitochondria, Hippocampus

## Abstract

Neuronal mitochondria play important roles beyond ATP generation, including Ca^2+^ uptake, and therefore have instructive roles in synaptic function and neuronal response properties. Mitochondrial morphology differs significantly between the axon and dendrites of a given neuronal subtype, but in CA1 pyramidal neurons (PNs) of the hippocampus, mitochondria within the dendritic arbor also display a remarkable degree of subcellular, layer-specific compartmentalization. In the dendrites of these neurons, mitochondria morphology ranges from highly fused and elongated in the apical tuft, to more fragmented in the apical oblique and basal dendritic compartments, and thus occupy a smaller fraction of dendritic volume than in the apical tuft. However, the molecular mechanisms underlying this striking degree of subcellular compartmentalization of mitochondria morphology are unknown, precluding the assessment of its impact on neuronal function. Here, we demonstrate that this compartment-specific morphology of dendritic mitochondria requires activity-dependent, Ca^2+^ and Camkk2-dependent activation of AMPK and its ability to phosphorylate two direct effectors: the pro-fission Drp1 receptor Mff and the recently identified anti-fusion, Opa1-inhibiting protein, Mtfr1l. Our study uncovers a signaling pathway underlying the subcellular compartmentalization of mitochondrial morphology in dendrites of neurons in vivo through spatially precise and activity-dependent regulation of mitochondria fission/fusion balance.

## Abstract (web summary)

The mechanisms regulating mitochondrial architecture in neurons remain unclear. The authors report that in dendrites, mitochondria structure is specified by the CAMKK2-AMPK pathway through compartment-specific and activity-dependent levels of fission.

## Introduction

Neurons are among the largest, most complex and highly polarized cell types found in nature. The two main neuronal compartments, dendrites and the axon, differ dramatically in their overall architecture, morphology and functional properties. This striking degree of polarization requires differential targeting of mRNA and proteins, differences in organelle composition, such as specific classes of endosomes, as well as compartment-specific organelle morphology and dynamics^1–5^. Two of the most abundant and complex organelles found in neurons, mitochondria and the endoplasmic reticulum, display a remarkable degree of morphological specialization between axonal and dendritic compartments^5–11^: in the axon of long-projecting mammalian cortical pyramidal neurons (PNs), mitochondria are small (~1 micron in length) and selectively localized to presynaptic boutons, whereas in the dendrites of the same neurons, mitochondria are long and tubular, filling a large fraction of the dendritic volume.

This high degree of polarization not only applies to these two broad neuronal compartments, but also to sub-compartments within dendrites. Our recent work has identified a dramatic sub-cellular compartmentalization of mitochondrial morphology within the dendrites of CA1 PNs of the hippocampus in vivo^12^. Unlike other classes of PNs, such as cortical layer 2/3 PNs^7^, dendritic mitochondria of CA1 PNs are fragmented in the dendritic compartments proximal to the soma (basal and apical oblique dendrites) but are significantly more tubular and occupy a larger fraction of the distal apical tuft compartment^12^. Interestingly, this subcellular compartmentalization of mitochondrial morphology corresponds directly to the spatial segregation of presynaptic inputs received by CA1 PNs: basal dendrites in stratum oriens (SO) and apical oblique dendrites in the stratum radiatum (SR) receive presynaptic inputs from intra-hippocampal axons originating from CA2/3 PNs, while distal apical tuft dendrites in the stratum lacunosum moleculare (SLM) receive extra-hippocampal inputs from the entorhinal cortex (EC).

Mitochondrial morphology in dendrites of pyramidal neurons was previously shown to be regulated by synaptic activity such that neuronal depolarization increased dendritic mitochondria fission through Ca^2+^-dependent activation of the small-GTPase Dynamin-related protein 1 (Drp1)^13–16^. However, most of these results were obtained in vitro and therefore their relevance for mitochondrial morphology of specific neuronal subtypes in vivo is largely unknown. In addition, Drp1 was recently shown to also be involved in regulating endocytosis in neurons^17,18^, obscuring the interpretation of these earlier results and leaving the molecular mechanisms whereby neuronal depolarization and/or synaptic activity could regulate dendritic mitochondrial morphology in vivo unresolved. Drp1 is a cytoplasmic dynamin-like small GTPase recruited to the outer mitochondrial membrane (OMM) by so-called Drp1 ‘receptors’ to promote constriction and fission. These Drp1-receptors include mitochondrial fission factor (Mff), fission 1 (Fis1), and mitochondrial dynamics proteins of 49 and 51 kDA (MiD49/MiD51)^19–21^, with Mff being the most dominant and universal Drp1 receptor in mammalian cells including in neurons.

AMP-activated kinase (AMPK, also called Protein kinase AMP-activated or Prka) is a heterotrimer composed of a catalytic α subunit, an adaptor subunit β and the γ subunit that binds to AMP/ADP. In most cells, AMPK plays a central role as a metabolic sensor activated when ATP levels drop in cells (i.e., when AMP/ADP levels increase). Once catalytically activated, AMPK phosphorylates many downstream substrates involved in restoring ATP levels and/or decreasing activity of pathways that consume significant levels of ATP such as protein synthesis^22,23^. In the context of metabolic stress, AMPK promotes mitochondrial fragmentation by directly phosphorylating Mff^24^ and Mtfr1l^25^, a newly characterized regulator of mitochondrial morphology. In mammalian neurons, AMPK is not only a metabolic sensor but also an activity-regulated kinase being phosphorylated and catalytically activated by Calcium/calmodulin dependent protein kinase kinase 2 (Camkk2), which is robustly induced by increase in intracellular Ca^2+^ levels following opening of voltage-gated Ca^2+^ channels (VGCC) induced by neuronal depolarization and, or activation of N-methyl D-aspartate (NMDA) receptors^12,26,27^. We have recently shown that Aβ42 oligomers trigger overactivation of Camkk2 leading to excessive activation of AMPK which phosphorylates Mff to trigger dendritic mitochondrial fission in apical tufts (SLM) of CA1 PNs dendrites in vivo^12^.

However, the physiological, developmental, and molecular mechanisms underlying this striking degree of mitochondrial compartmentalization in dendrites of CA1 PNs in vivo are largely unknown, preventing the exploration of its functional significance. Here, we report that the compartmentalized morphology of dendritic mitochondria characterizing CA1 PNs in vivo is present early in development, refined during postnatal maturation of CA1 PNs dendrites and requires activity-dependent and input-specific regulation of the Camkk2-AMPK kinase dyad. Here, we demonstrate that the relationship between neuronal activity, dendritic Ca^2+^ dynamics and mitochondrial morphology differs across the dendritic domains of CA1 PNs in vivo: in proximal basal and apical oblique dendrites, but not in apical tuft dendrites, high levels of activity- and Ca^2+^-dependent, Camkk2-dependent AMPK activity drives high levels of phosphorylation of two of its direct effectors, Mff and Mtfr1l, promoting mitochondrial fission and opposing fusion, respectively. Our 2-photon Ca^2+^ imaging, loss-of-function and rescue experiments performed in vivo identify a molecular pathway regulating the compartment-specific morphology of dendritic mitochondria of CA1 PNs through local, activity-dependent, control of mitochondrial fission/fusion balance.

## Results

### CA1 hippocampal pyramidal neurons display sub-cellular, compartment specific dendritic mitochondria morphology in vivo

**Fig. 1:**
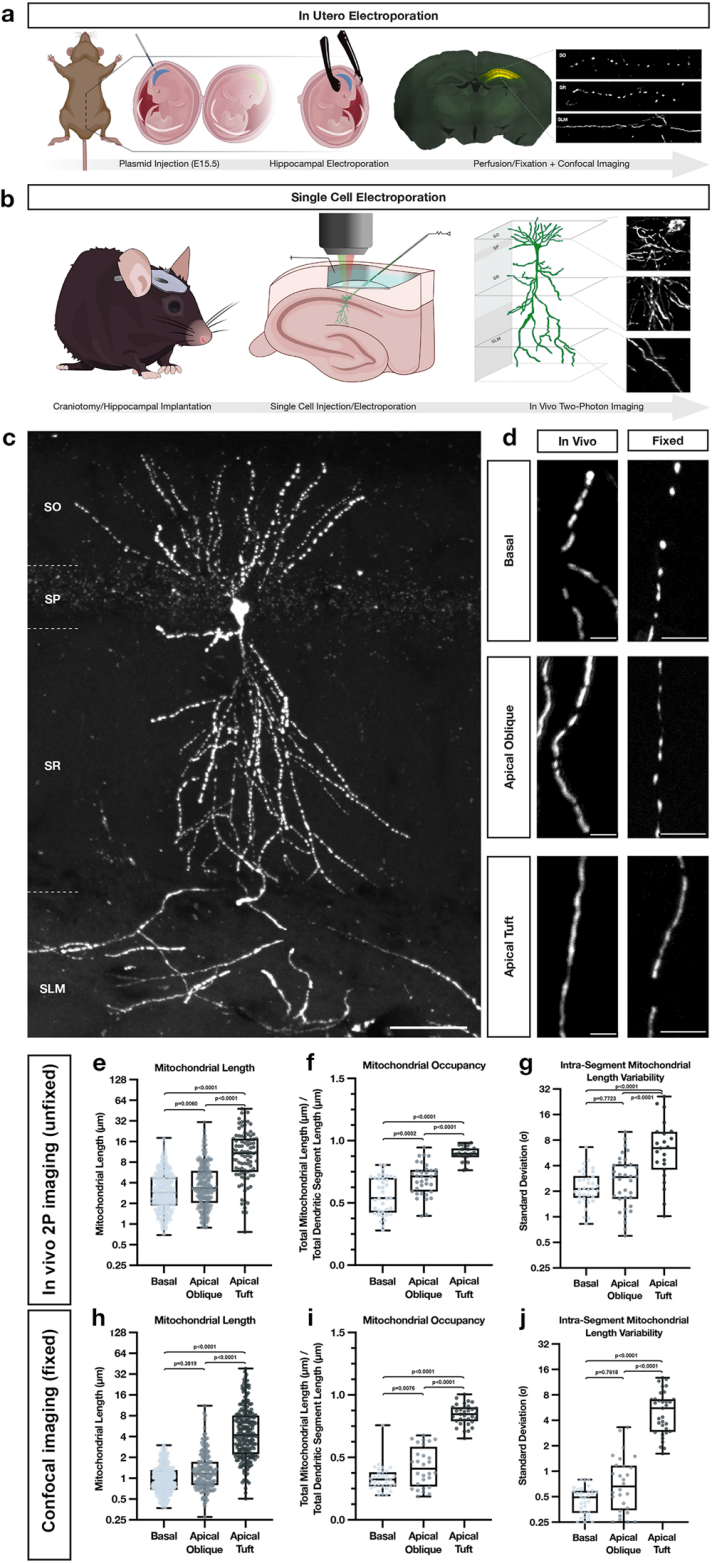
Mitochondria display highly compartmentalized, layer-specific, morphology in dendrites of CA1 PNs in vivo. **a** Schematic of *in utero* electroporation (IUE) procedure used to sparsely express fluorescent reporters, cDNAs or shRNAs in CA1 PNs of the hippocampus. **b** Schematic of single cell electroporation (SCE), used to express fluorescent reporters into single adult CA1 pyramidal neurons in the hippocampus. This method allows for rapid expression of plasmid DNA and in vivo two-photon visualization of individual neurons in an adult anesthetized mouse in vivo. Portions of (**a**) and (**b**) were created with BioRender.com. **c** Representative image of a single CA1 PN expressing a mitochondrial matrix reporter (mt-YFP) via SCE following fixation and re-imaged using confocal microscopy on a single vibratome section. Scale bar, 50 μm. **d** High-magnification representative images of dendrites from the three hippocampal compartments—basal (SO), apical oblique (SR), and apical tuft (SLM)—expressing a mitochondrial matrix marker (mt-YFP or mt-DsRed) imaged either in vivo using two-photon microscopy (left) or post-fixation with confocal microscopy (right). Scale bar, 5 μm. **e**–**g** Quantification of individual mitochondrial length (**e**), mitochondrial segment occupancy (**f**), and intra-segment mitochondrial length variability (**g**) from basal, apical oblique, and apical tuft dendrites captured in vivo. (Basal: n = 422 mitochondria; n = 37 dendritic segments; mean length = 3.621 μm ± 0.124 (SEM); mean occupancy = 56.11% ± 2.49% (SEM); mean intra-segment variability = 2.420 μm ± 0.198 (SEM); Apical Oblique: n = 263 mitochondria; n = 35 dendritic segments; mean length = 4.719 μm ± 0.249 (SEM); mean occupancy = 68.82% ± 2.20% (SEM); mean intra-segment variability = 3.185 μm ± 0.370 (SEM); Apical Tuft: n = 90 mitochondria; n = 23 dendritic segments; mean length = 13.02 μm ± 1.041 (SEM); mean occupancy = 89.26% ± 1.24% (SEM); mean intra-segment variability = 8.018 μm ± 6.359 (SEM)). **h**–**j** Quantification of individual mitochondrial length (**h**), mitochondrial segment occupancy (**i**), and intra-segment mitochondrial length variability (**j**) from basal, apical oblique, and apical tuft dendrites following fixation. (Basal: n = 313 mitochondria; n = 32 dendritic segments; mean length = 1.065 μm ± 0.029 (SEM); mean occupancy = 33.01% ± 1.84% (SEM); mean intra-segment variability = 0.4736 μm ± 0.030 (SEM); Apical Oblique: n = 184 mitochondria; n = 28 dendritic segments; mean length = 1.569 μm ± 0.104 (SEM); mean occupancy = 42.38% ± 2.98% (SEM); mean intra-segment variability = 0.9169 μm ± 0.149 (SEM); Apical Tuft: n = 226 mitochondria; n = 33 dendritic segments; mean length = 6.562 μm ± 0.437 (SEM); mean occupancy = 84.19% ± 1.44% (SEM); mean intra-segment variability = 5.695 μm ± 0.552 (SEM)). p values are indicated in the figure following a one way ANOVA with Sidak’s multiple comparisons test. Data are shown as individual points on box plots with 25^th^, 50^th^ and 75^th^ percentiles indicated with whiskers indicating min and max values.

To visualize mitochondrial morphology in adult CA1 PNs in vivo in unfixed and fixed conditions, we used two complementary electroporation techniques: in utero electroporation (IUE; Fig. 1a) and in vivo single cell electroporation (SCE; Fig. 1b). Both techniques enable the labeling of mitochondria to visualize their morphology with enough sparsity for optical isolation of single CA1 PNs in vivo. The SCE approach has the advantage of enabling in vivo live 2-photon imaging of mitochondria in an anesthetized adult mouse, preventing any potential artifact of fixation which can affect mitochondria morphology^28^. IUE also allows for sparse expression of mitochondrial markers in vivo and is coupled with fixation and imaging with confocal microscopy on brain slices. In both techniques, we co-electroporated a cytoplasmic fluorescent protein (tdTomato, mGreenLantern or mTagBFP2) and a mitochondrial matrix-targeted fluorescent protein (mt-YFP, mt-DsRed or mt-mTAGBFP2) allowing us to visualize single mitochondrial matrices in optically isolated dendrites in developing or adult CA1 PNs.

As previously reported^12^, quantitative measurement of mitochondrial matrix size and dendritic occupancy following fixation of IUE CA1 PNs shows a remarkable degree of subcellular compartmentalization corresponding with spatially restricted afferents arriving onto distinct portions of the dendritic arbor (Fig. 1c, d, h–j). In basal dendrites (SO) of CA1 PNs located, mitochondria are short (1.065 μm ± 0.029), occupy a third of the dendritic segments (33.01% ± 1.84%), and are uniform in length (mean intra-segment variability: 0.4736 μm ± 0.030). In apical oblique dendrites located in SR, mitochondria are also short, though nearly 1.5 times longer than in SO (1.569 μm ± 0.104), occupy just under half of the dendritic processes (42.38% ± 2.98%), and are rather uniform (mean intra-segment variability: 0.9169 μm ± 0.149). In apical tuft dendrites located in SLM, mitochondria are significantly more elongated (6.562 μm ± 0.437) than in SO or SR, occupy a larger proportion of dendritic segments (84.19% ± 1.44%), and vary significantly more in size within individual dendritic segments (mean intra-segment variability, 5.695 μm ± 0.552).

To exclude the possibility that this compartment-specific morphology of dendritic mitochondria could be the result of fixation^28^, we turned to SCE to image dendritic mitochondria of CA1 PNs in living mice. Two to three days following SCE with a cytoplasmic and mitochondrial matrix marker, mice were placed under isoflurane anesthesia while individual dendritic segments from all three compartments—basal (SO), apical oblique (SR), and apical tuft (SLM)—were imaged using in vivo 2-photon microscopy. Despite different imaging conditions, quantitative measurements of mitochondrial size, occupancy, and intra-segment variability show conserved relationships between all three compartments with both approaches (Fig. 1d, e–g). These results demonstrate that the striking degree of compartmentalization of dendritic mitochondria characterizing CA1 PNs in vivo is not the result of fixation artifacts.

Because the above observations were made using mitochondrial matrix-targeted fluorescent reporters, we repeated the IUE experiments, this time expressing fluorescent proteins targeted to both the mitochondrial matrix (mt-mTAGBFP2 or mt-YFP) and the outer mitochondrial membrane (OMM; ActA-mCherry-HA). Quantification of both mitochondrial size and occupancy using both OMM and matrix markers confirmed the above observations. Interestingly, however, values obtained with the OMM marker were significantly longer in all three compartments than their respective matrix measurements, while still maintaining significantly compartmentalized differences (Fig. S1a–c).

This result indicates that individual mitochondria defined by OMM markers can contain fragmented matrix sub-volumes, which can be challenging to resolve using diffraction-limited light microscopy. Previous work has reported that in neuronal dendrites, the IMM can undergo repetitive constriction events, termed CoMIC, independently of OMM membrane dynamics^29^. In order to determine the ultrastructural features of mitochondria in CA1 PNs dendrites in vivo, and to confirm that dendritic mitochondria in SLM and SR adopt distinct morphologies using an independent imaging approach, we took advantage of a serial EM dataset previously published for connectomic analysis^30^. In this dataset, mitochondria located in the apical dendrites of CA1 PNs located in SLM and SR were reconstructed in 3D (Fig. S1d–h) and our analysis confirms striking differences in mitochondria morphology between these two dendritic compartments. In SR, mitochondria adopt a unique morphology with thin constrictions (often <100 nm in diameter; arrow in Fig. S1e) and bulging of the matrix (arrowheads in Fig. S1e). This unique ultrastructural feature is significantly less frequent in mitochondria found in SLM which presents more uniformly tubular morphologies (see multiple examples in Fig. S1h). This morphological difference results in a significant reduction in the volume of dendrite occupied by mitochondria in SR compared to SLM (Fig. S1i).

Overall, our light and serial electron microscopy approaches converge to demonstrate that mitochondrial morphology is strikingly compartmentalized in CA1 PNs in vivo with highly fused, elongated and tubular morphology in SLM and progressively more fragmented matrix compartments in SR and SO.

### Compartment-specific dendritic mitochondria morphology is present early in the development of CA1 PNs in vivo but is absent in vitro

To determine the developmental timeframe for the emergence of compartmentalized mitochondria morphology described above in adult CA1 PNs in vivo, we collected in utero electroporated brains at postnatal days seven, ten, fourteen and twenty-one (P7, P10, P14 and P21). At all stages of development, we observed that mitochondria are significantly shorter in the basal dendrites located in SO than in the apical tuft dendrites located in SLM (Fig. S2a). Interestingly, while this compartment-specific mitochondrial morphology is clearly present at each developmental timepoint, mitochondria increase in both size (Fig. S2b) and dendritic occupancy (Fig. S2c) in both domains throughout maturation and reach adult-like values by P21.

To determine whether compartmentalized mitochondrial morphology characterizing CA1 PNs in vivo is regulated by cues intrinsic to the dendrites or cues extrinsic to CA1 PNs, we performed IUE to label mitochondria of CA1 PNs at E15.5 as above but at E18.5, the hippocampi of electroporated embryos were collected, enzymatically dissociated, and maintained in 2-dimensional (2D) cultures in vitro (Fig. S3a, b). Following culture for ten, fourteen or eighteen days in vitro (DIV10, 14, 18), we observed a near complete loss of compartmentalization of mitochondria morphology with either no significant difference in mitochondrial length or occupancy (at DIV10 and 18), or opposite results to those found in vivo: mitochondria were slightly but significantly longer with higher occupancy values in the proximal dendrites compared to distal dendrites (14DIV) (Fig. S3c, d). These results suggest that factors extrinsic to CA1 PNs, potentially including local activity patterns or input-specific molecular cues, might drive the formation of the compartment-specific mitochondrial morphology characterizing CA1 PNs in vivo.

### Dendritic Ca^2+^ transients show compartment-specific frequency and amplitude in vivo

**Fig. 2:**
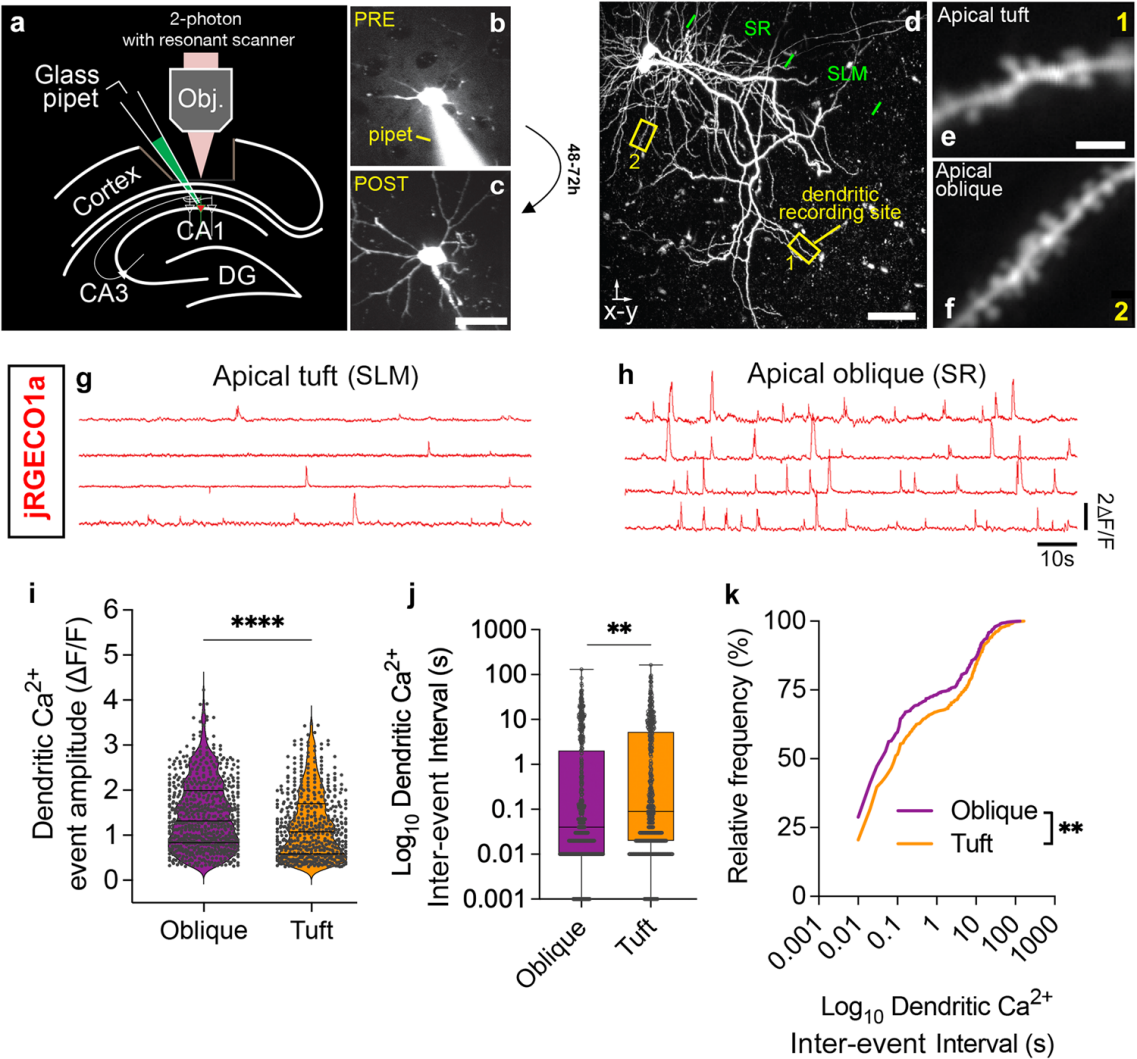
Two-photon imaging in CA1 PNs in vivo reveals lower amplitude and frequency of Ca^2+^ transients in apical tuft dendrites compared to apical oblique dendrites. **a**–**c** Schema of single cell electroporation (SCE^66^) in vivo using visually-guided somatic targeting (**b**, **c**) in CA1 PNs with a glass pipet containing a fluorescent dye (Alexa 488 in (**b**)) and a mix of plasmids encoding fluorescent protein (Venus in example shown in (**c**)). **d**–**f** Maximal z-projection following in vivo 2-photon imaging of two CA1 PNs neurons expressing genetically-encoded Ca^2+^ sensor jRGECO1a through SCE (**d**) showing the full extent of dendrites from the basal (SO) all the way to apical obliques (SR) and the apical tuft (SLM). Example of high magnification recording sites in the apical tuft (SLM in (**e**) – corresponding to box 1 in (**d**)) and in the apical oblique (SR in (**f**) – corresponding to box 2 in (**d**)). Individual dendritic spines can be observed. **g**, **h** Representative traces from jRGECO1a fluorescence showing individual Ca^2+^ transients isolated from the dendritic shaft of segments in the apical tuft (SLM in (**g**)) and the apical oblique domains (SR in (**h**)). Quantification of dendritic Ca^2+^ transient amplitude (**i**) and inter-event intervals (inverse of frequency) shown as box plots (**j**) or cumulative frequency (**k**). Statistical analysis: (**i**) **** p < 0.0001 according to two-tailed, Mann-Whitney non-parametric test. Data shown as violin plot with individual points shown. **j**, **k** ** p = 0.003 according to Kolmogorov-Smirnov non-parametric test. n = 632 events for oblique (from 51 dendritic segments imaged) and n = 508 events for tuft (from 47 dendritic segments imaged) in total of 11 CA1 PNs. Scale bars: 20 microns in (**b**) and (**c**); 50 microns in (**d**); 2 microns in (**e**) and (**f**). Data are shown as individual points on box plots with 25^th^, 50^th^ and 75^th^ percentiles indicated with whiskers indicating min and max values.

We previously demonstrated that mitochondrial fission/fusion dynamics are regulated by the Ca^2+^-dependent Camkk2-AMPK signaling pathway in CA1 PNs^12^. Therefore, we hypothesized that the striking compartmentalization of mitochondrial morphology described above in vivo could be regulated by differential dynamics of cytoplasmic Ca^2+^ dynamics between apical tuft and more proximal dendritic compartments in CA1 PNs in vivo. To explore this, we performed a combination of (1) SCE (Fig. 2a–c) of a plasmid expressing the red-shifted Ca^2+^ indicator jRGECO1a in adult CA1 PNs coupled with (2) in vivo Ca^2+^ imaging (using jRGECO1a) in the apical tuft (SLM) and apical oblique dendritic segments (SR) using 2-photon microscopy (Fig. 2d–f). We recorded dendritic Ca^2+^ transients (Fig. 2g, h) and observed significantly higher amplitude (Fig. 2i) and higher frequency (lower inter-event intervals; Fig. 2j, k) of Ca^2+^ transients in apical oblique (SR) compared to apical tuft (SLM) dendritic segments in vivo.

### Neuronal activity and domain-specific synaptic inputs regulate the formation of compartmentalized mitochondrial morphology of CA1 PNs in vivo

The results above, coupled with previous results obtained in vitro suggesting that synaptic activity induces local Drp1-dependent mitochondrial fission events in the context of synaptic plasticity^13^, led us to test if the compartmentalized morphology of dendritic mitochondria observed in CA1 PNs in vivo is regulated by neuronal activity. We performed two classes of experiments in order to (1) cell-autonomously reduce CA1 PNs spiking activity or (2) cell-autonomously reduce the fraction of CA3 inputs making synapses onto CA1 PNs.

**Fig. 3:**
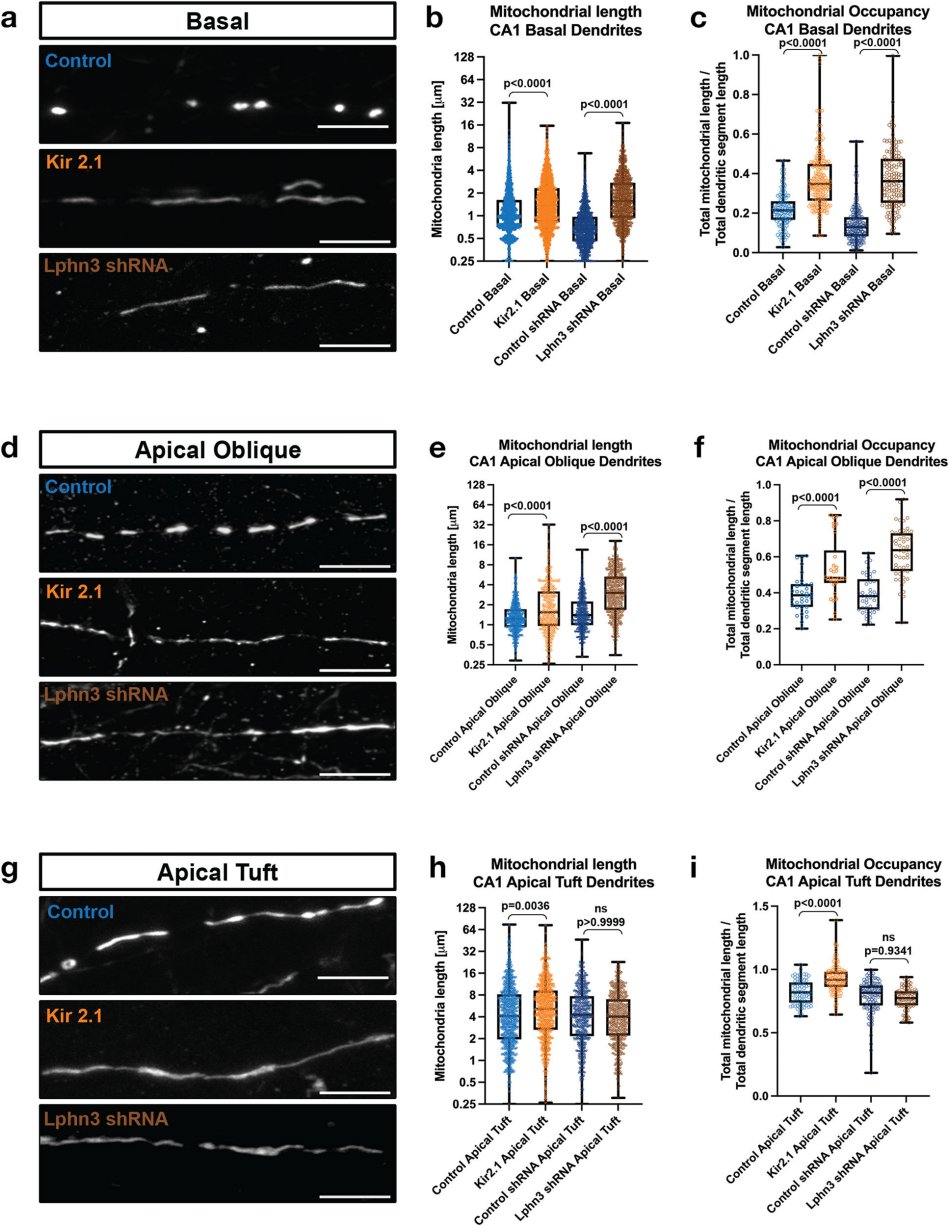
Neuronal activity regulates mitochondrial size in a compartment specific manner in CA1 pyramidal neurons in vivo. **a**–**i** High magnification representative images of mitochondrial morphology within isolated secondary or tertiary hippocampal CA1 (**a**) basal, (**d**) apical oblique, and (**g**) apical tuft dendrites in which a mitochondrial matrix-targeted fluorescent protein (mt-YFP) was IUE along with either a control plasmid (pCAG tdTomato) or pCAG Kir2.1-T2A-tdTomato), or either control shRNA or Lphn3 shRNA plasmid. Quantification of mitochondrial length and occupancy in the (**b**) and (**c**) basal, (**e**) and (**f**) apical oblique, and (**h**) and (**i**) apical tuft dendritic compartments following Kir2.1 over-expression or shRNA-mediated knockdown of Lphn3. Quantification of mitochondrial length (**b**), (**e**), and (**h**) and mitochondrial occupancy (**c**, **f** and **i**) in basal dendrites (**b**, **c**), apical oblique (**e**, **f**) and apical tuft (**h**, **i**) demonstrates that both Kir2.1 over-expression and Lphn3 knockdown significantly increases mitochondrial length and occupancy in basal (**b**, **c**) and apical oblique dendrites (**e**, **f**). Control_tdTomato-basal_ = 127 segments, 2154 mitochondria, mean length = 1.42 µm ± 0.03 µm (SEM), mean occupancy = 21.8% ± 0.9% (SEM); Control_tdTomato-apical oblique_ = 34 segments, 748 mitochondria, mean length = 1.49 µm ± 0.04 µm, mean occupancy = 39.4% ± 1.0%; Control_tdTomato-apical tuft_ = 75 segments, 1036 mitochondria, mean length = 6.48 µm ± 0.23 µm, mean occupancy = 81.9% ± 1.1%; Kir2.1_tdTomato-basal_ = 206 segments, 2278 mitochondria, mean length = 1.87 µm ± 0.03 µm, mean occupancy = 37.8% ± 1.0%; Kir2.1_tdTomato-apical oblique_ = 30 segments, 435 mitochondria, mean length = 2.54 µm ± 0.14 µm, mean occupancy = 53.5% ± 2.9%; Kir2.1_tdTomato-apical tuft_ = 128 segments, 965 mitochondria, mean length = 7.19 µm ± 0.22 µm, mean occupancy = 92.7% ± 1.0%; Control_shRNA-basal_ = 169 segments, 1344 mitochondria, mean length = 0.81 µm ± 0.01 µm, mean occupancy = 13.8% ± 0.6%; Control_shRNA-apical oblique_ = 45 segments, 633 mitochondria, mean length = 1.84 µm ± 0.05 µm, mean occupancy = 39% ± 1.6%; Control_shRNA-apical tuft_ = 106 segments, 617 mitochondria, mean length = 5.81 µm ± 0.20 µm, mean occupancy = 78.6% ± 1.2%; Lphn3_shRNA-basal_ = 129 segments, 1169 mitochondria, mean length = 2.11 µm ± 0.04 µm, mean occupancy = 37.4% ± 1.3%; Lphn3_shRNA-apical oblique_ = 53 segments, 573 mitochondria, mean length = 3.92 µm ± 0.12 µm, mean occupancy = 62.5% ± 1.9%; Lphn3_shRNA-apical tuft_ = 84 segments, 561 mitochondria, mean length = 5.1 µm ± 0.16 µm, mean occupancy = 75.2% ± 0.9%. p values are indicated in the figure following a Kruskal-Wallis test. Data are shown as individual points on box plots with 25^th^, 50^th^ and 75^th^ percentiles indicated with whiskers indicating min and max values. Scale bar, 5 μm.

First, we performed IUE to express the inward rectifying potassium channel 2.1 (Kir2.1) which hyperpolarizes neurons to ~−90mV, drastically reducing their excitability and ability to fire action potentials^31,32^. Strikingly, Kir2.1 overexpression in CA1 PNs in vivo through development strongly reduced the compartment-specific differences in mitochondria morphology. Dendritic mitochondria in Kir2.1-expressing CA1 PNs became significantly longer and occupied a higher percentage of the dendrites in SO (basal dendrites, Fig. 3a–c), SR (apical obliques, Fig. 3d–f), and SLM (apical tufts, Fig. 3g–i) compared to control CA1 PNs. This result demonstrates that neuronal activity is required for the proper maturation of compartmentalized morphology of dendritic mitochondria in CA1 PNs in vivo.

We next cell-autonomously reduced the number of synaptic inputs received by CA1 PNs in a dendritic compartment-specific manner by downregulating Latrophilin 3 (Lphn3, Fig. S4), a postsynaptic adhesion molecule previously shown to be required for the ability of CA3 axons to form ~50% of synapses onto dendrites of CA1 PNs specifically in the SO and SR compartments, but not for synapses made by EC axons in SLM^33^. This synapse-specific manipulation results in a reduction by ~50% of dendritic spine density in SR dendrites and a corresponding ~50% reduction in miniature or evoked excitatory potential synaptic currents (EPSC) in CA1 PNs^33^. We confirmed that shRNA-mediated knockdown of Lhpn3 (Fig. S4a, b) by IUE of CA1 PNs leads to ~50% reduction in spine density in SO but not in SLM (Fig. S4c, d).

Interestingly, this cell-autonomous reduction of the number of CA3 presynaptic inputs mediated by Lphn3 knockdown in CA1 PNs led to a striking and significant increase in mitochondria size and occupancy in dendritic compartments receiving CA3 inputs (SO and SR; Fig. 3a–f), while mitochondria morphology in the apical tuft (SLM) of the same CA1 PNs were unaffected (Fig. 3g–i). These results show that (1) local synaptic activity is a major regulator of mitochondria morphology in dendrites of CA1 PNs and (2) suggests that an activity-dependent signaling mechanism promotes the small mitochondria morphology in SO and SR in vivo, potentially by promoting mitochondria fission and/or inhibiting mitochondria fusion in basal and apical oblique dendrites but not in the apical tuft dendrites.

### Acute blockage of neuronal activity affects mitochondrial morphology in adult CA1 PNs in vivo

**Fig. 4:**
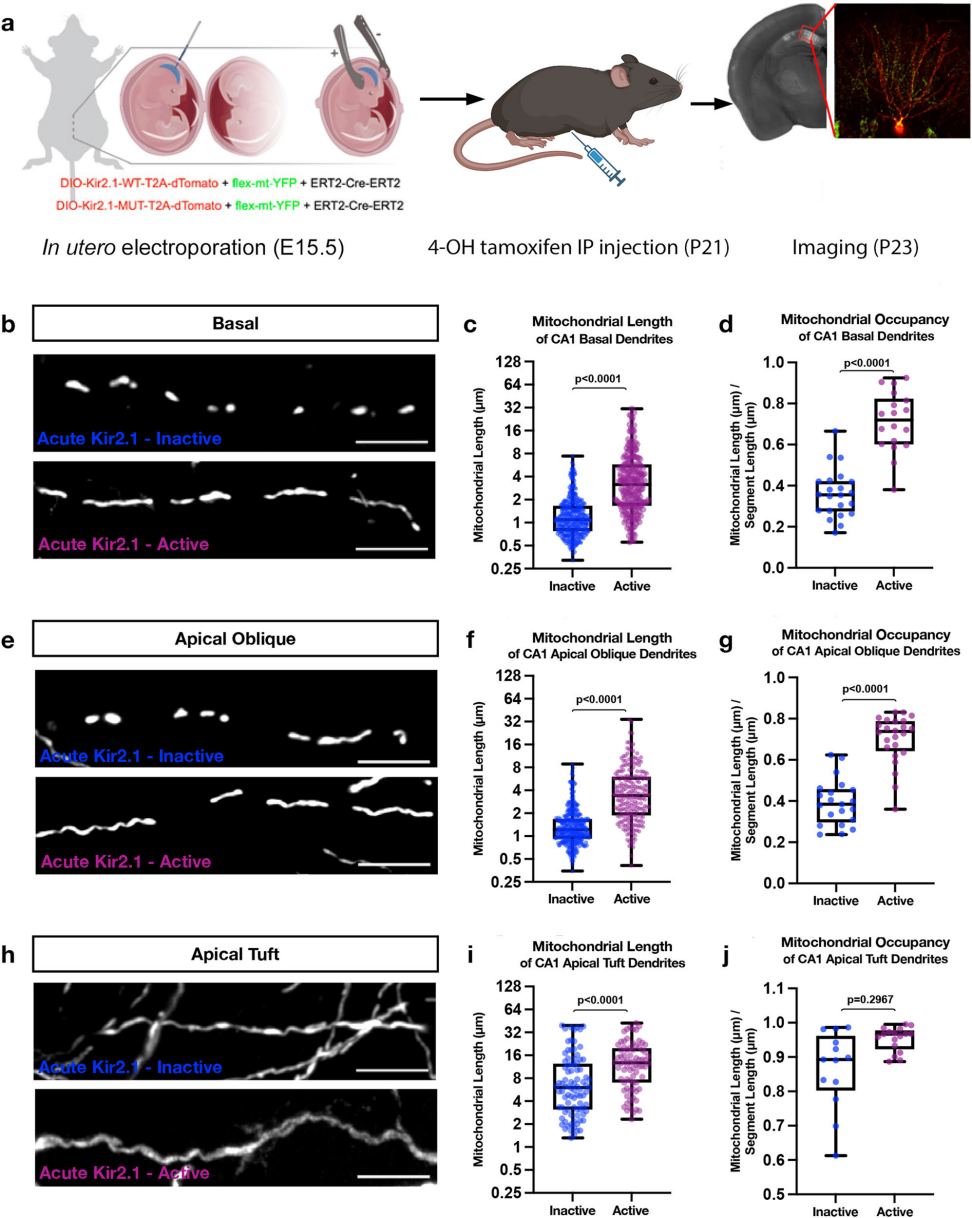
Acute silencing of CA1 PNs at P21 leads to elongated mitochondria across the dendritic arbor. **a** Schematic representation of our experimental timeline with electroporation of an inducible ERT2-Cre-ERT2 along with either a mutated (inactive) or wild type (active) DIO-Kir2.1-T2A-dTomato and a flex-mt-YFP occurred on E15.5. Mice were then injected with 4-hydroxytamoxifen (4-OHT) at P21 and perfused, sliced, and imaged on P23. Low magnification image is of an induced Kir2.1-inactive cell with mt-YFP. Representative high magnification images of mitochondrial morphology within hippocampal CA1. Portions of (**a**) were created with BioRender.com. **b** Basal, **e** apical oblique, and **h** apical tuft dendrites. Quantification of the mitochondrial length and occupancy in the (**c**, **d**) basal, (**f**, **g**) apical oblique, and (**i**, **j**) apical tuft dendritic compartments reveal a significant increase in mitochondrial length (**c**) and occupancy (**d**) in basal dendrites when mature cells are silenced. A similar effect is seen in the apical tuft (**f**, **g**), however, while acute silencing leads to a significant increase in the length of mitochondria in the apical tuft (**i**) it does not alter the total proportion of the branch occupied by mitochondria (**j**). Basal Dendrites (Acute Kir2.1-inactive: n = 22 dendritic segments, 349 individual mitochondria, mean length = 1.403μm ± 0.307(SEM), mean occupancy = 36.12% ± 5.843%; Acute Kir2.1-active n = 19 dendritic segments, 218 individual mitochondria, mean length = 4.793 μm ± 0.055 (SEM), mean occupancy = 71.07% ± 5.69%.) Apical Oblique Dendrites (Acute Kir2.1-inactive: n = 21 dendritic segments, 341 individual mitochondria, mean length = 1.548 μm ± 0.303 (SEM), mean occupancy = 39.32% ± 4.278%; Acute Kir2.1-active: n = 24 dendritic segments, 232 individual mitochondria, mean length = 4.797 μm ± 0.016 (SEM), mean occupancy = 70.12% ± 5.827%.) Apical Tuft Dendrites (Acute Kir2.1-Mut: n = 13 dendritic segments, 89 individual mitochondria, mean length = 9.670 μm ± 1.109 (SEM), mean occupancy = 86.52% ± 7.34%; Acute Kir2.1-Mut: n = 17 dendritic segments, 76 individual mitochondria, mean length = 14.40 μm ± 1.04 (SEM), mean occupancy = 95.27% ± 12.15%.) p values are indicated in the figure following a one way ANOVA with Sidak’s multiple comparisons test. Data are shown as individual points on box plots with 25^th^, 50^th^ and 75^th^ percentiles indicated with whiskers indicating min and max values. Scalebar, 5 μm.

Next, we asked whether acute manipulation of neuronal activity in mature CA1 PNs had the same impact as developmental manipulations on the compartmentalization of dendritic mitochondria structure. To test this, we electroporated CA1 PNs with a tamoxifen-inducible form of Cre recombinase (ERT2-CRE-ERT2^34^), along with a Cre-dependent expression plasmid encoding either a mutated (inactive) or wild type (active) Kir2.1-T2A-tdTomato (Fig. 4a). We induced Kir2.1 and tdTomato expression on P21, when mitochondrial compartmentalization is fully established, using intraperitoneal (IP) injections of 4-hydrotamoxifen (4-OHT) and perfused brains two days later (P23; Fig. 4a). 4-OHT successfully induced expression of tdTomato (Fig. 4a). Our results show that inducing acute neuronal hyperpolarization in mature CA1 PNs in vivo significantly increases mitochondria length (Fig. 4b, c, e, f) and mitochondrial occupancy in the proximal dendritic compartments (SO and SR; Fig. 4d, g). In the apical tuft, the mitochondria in cells expressing the active Kir2.1 were significantly longer than cells with the inactive form, however, the increase in total occupancy of mitochondria in these distal branches did not reach significance (Fig. 4h–j). Together these results demonstrate that depolarization of dendritic branches is required to maintain the compartmentalization of dendritic mitochondria structure characterizing CA1 PNs in vivo.

### Activity-dependence of mitochondrial fission-fusion rate in dendrites of pyramidal neurons

**Fig. 5:**
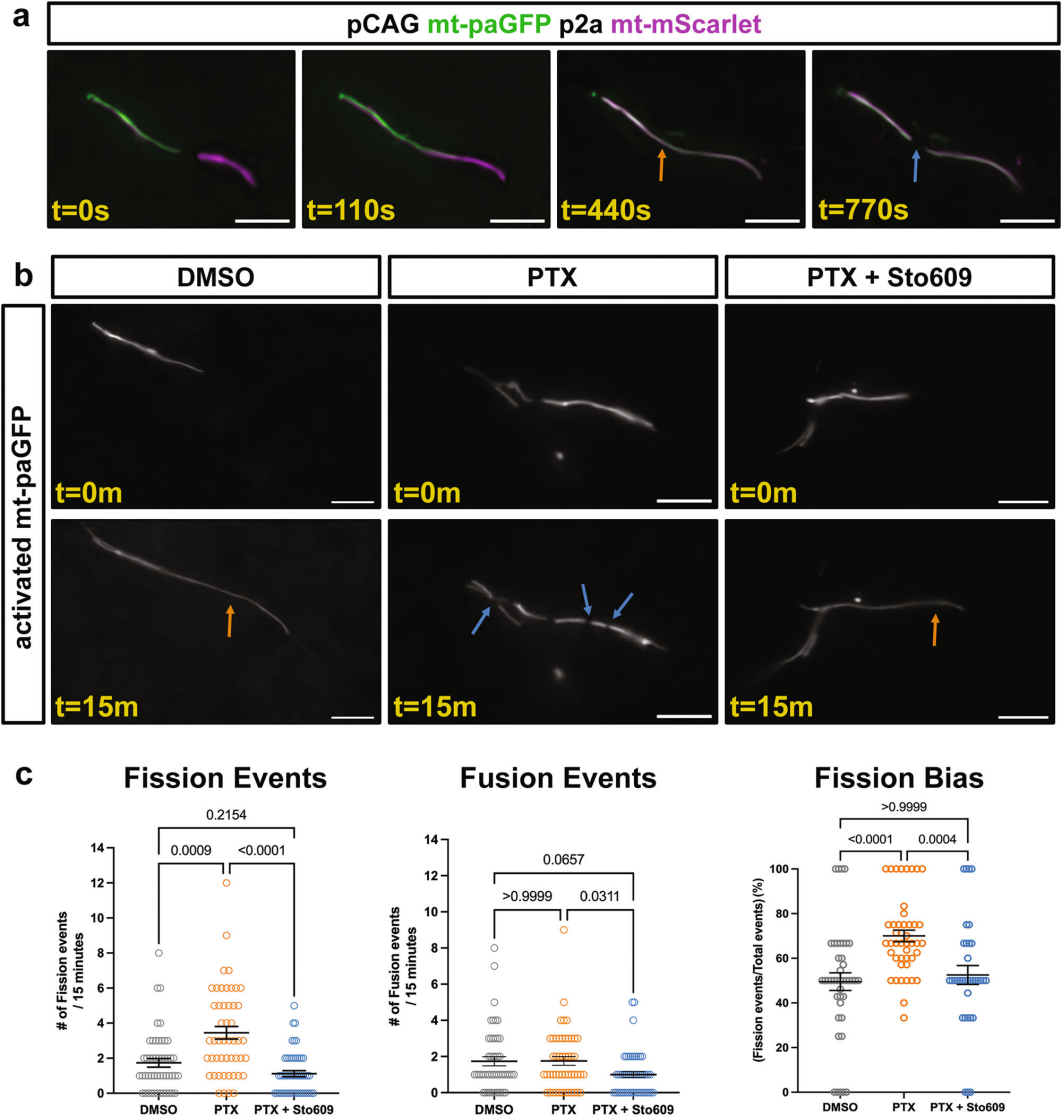
Neuronal activity induces mitochondrial fission that is blocked by the Camkk2 inhibitor STO609. **a** Mitochondrial fission and fusion was visualized in dendrites of primary cortical pyramidal neurons electroporated with a plasmid encoding matrix targeted photo-activatable GFP (mt-paGFP) and matrix targeted mScarlet (mt-mScarlet). At 14DIV timelapse imaging was performed following photoactivation of a small subset of dendritic mitochondria. The orange arrow points to a fusion event followed by a fission event (blue arrow). **b** Images of mt-paGFP following the indicated treatment at 0 and 15 minutes after photo-activation. Orange arrows point to fusion events, blue arrows to fission events. **c** Quantification of fission and fusion rates for dendritic photo-activated mitochondria in the indicated conditions demonstrating that activity increases the rate of mitochondrial fission through a Camkk2 dependent pathway. DMSO = 49 photoactivated dendritic ROIs from 11 neurons, 1.74 ± 0.24 fission and 1.74 ± 0.26 fusion events per 15 minutes (mean ± SEM). PTX = 49 photoactivated dendritic ROIs from 11 neurons, 3.45 ± 0.36 fission and 1.76 ± 0.24 fusion events per 15 minutes (mean ± SEM). PTX + STO609 = 52 photoactivated dendritic ROIs from 13 neurons, 1.12 ± 0.17 fission and 1.0 ± 0.17 fusion events per 15 minutes (mean ± SEM). p values are indicated in the figure following Kruskal-Wallis tests. Data are shown as individual points with mean ± SEM. Scale bar, 5 μm.

To independently assess the impact of neuronal activity on dendritic mitochondrial morphology, we developed an assay to measure the rates of mitochondrial fission and fusion upon manipulation of neuronal activity in vitro. In order to increase neuronal activity in a physiologically relevant manner, we treated cultured neurons with picrotoxin (PTX), a well-established paradigm to block GABAergic inhibition thus increasing neuronal activity by promoting glutamatergic excitatory synaptic transmission in pyramidal neurons in vitro^35–37^. Following PTX application (50 µM), mitochondrial fission and fusion dynamics were assayed via photoactivation of a matrix-targeted photoactivatable GFP (mt-paGFP, Fig. 5a)^38^. At 14DIV, when mitochondrial morphology is already established, we observed balanced fission and fusion rates in photoactivated dendritic mitochondria of DMSO treated neurons (Fig. 5b, c). However, in the presence of PTX, the number of fission events almost doubled while fusion remained stable leading to a dramatic shift towards mitochondrial fission (DMSO = 49.5% ± 3.9% fission, PTX = 70% ± 2.6% fission) strongly arguing that increased neuronal activity drives mitochondrial fission (Fig. 5b, c). To determine if the observed activity-dependent increase in mitochondrial fission requires Camkk2 activation as we had shown previously in CA1 PNs^12^, we co-treated with STO609, a specific inhibitor of Camkk2^39,40^. Interestingly, STO609 completely blocked the PTX-induced increase in mitochondrial fission, thus maintaining balanced rates of mitochondrial fission and fusion (STO609 + PTX = 52.5% ± 4.2% fission, Fig. 5b, c). Taken together, these results argue that neuronal activity drives increased mitochondrial fission in a calcium-dependent manner.

### Compartmentalized mitochondrial morphology in CA1 PNs requires Camkk2 and AMPK in vivo

We and others previously showed that the kinase dyad Camkk2 and AMPK are regulated by neuronal activity since neuronal activity, activation of VGCCs by dendritic depolarization, or activation of NMDA receptors can activate AMPK in a Camkk2-dependent manner in cortical and hippocampal neurons^12,26,41^. Furthermore, we also demonstrated that Camkk2-dependent AMPK over-activation mediates excessive mitochondrial fission through direct phosphorylation of Mff in apical dendrites of CA1 PNs^12,24^. Therefore, we hypothesized that activity-dependent mitochondrial fission could regulate mitochondrial compartmentalization in a Camkk2-AMPK-dependent manner whereby low Ca^2+^-dependent Camkk2-AMPK activity in apical tufts of CA1 PNs leads to fusion-dominant mitochondrial elongation in SLM and higher Ca^2+^-dependent Camkk2-AMPK activity in basal and apical oblique dendrites leads to fission-dominant smaller mitochondrial morphology in SO and SR.

**Fig. 6:**
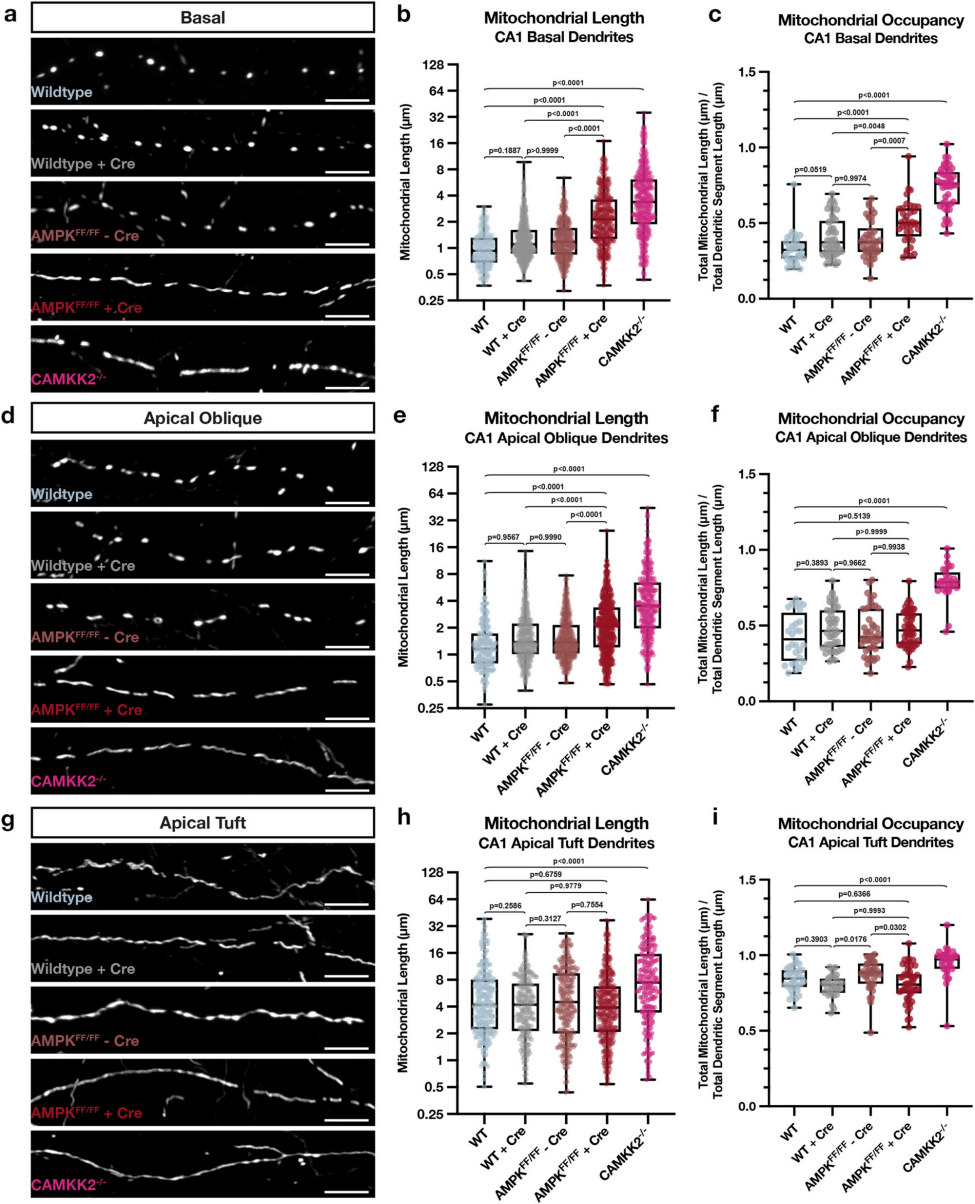
Camkk2 and AMPK are required for the compartment-specific mitochondrial morphology in CA1 PNs in vivo. **a**–**i** High magnification representative images of mitochondrial morphology within isolated secondary or tertiary hippocampal CA1 (**a**) basal, (**d**) apical oblique, and (**g**) apical tuft dendrites in which a mitochondrial matrix-targeted fluorescent protein (mt-YFP or mt-DsRed) and cell fill (tdTomato or mGreenLantern—not shown) were co-expressed by IUE with or without Cre recombinase (+/-Cre). AMPKα1^F/F^α2^F/F^ double conditional mice were *in utero* electroporated with the same mitochondrial markers/cell fills and either no Cre (AMPK^FF/FF^ - Cre) or Cre (AMPK^FF/FF^ + Cre). Camkk2^-/-^ constitutive knock-out mice were electroporated with the same mitochondrial markers/cell fills as above (Camkk2^-/-^). Quantification of mitochondrial length and occupancy in the (**b**, **c**) basal, (**e**, **f**) apical oblique, and (**h**, **i**) apical tuft dendritic compartments reveals a significant increase in mitochondrial length (**b**) and occupancy (**c**) in basal dendrites when knocking out AMPK or Camkk2 when compared to their controls, with Camkk2 KO having a much bigger effect (WT: n = 32 dendritic segments, 313 individual mitochondria, mean length = 1.065 μm ± 0.029 (SEM), mean occupancy = 33.01% ± 1.84%; WT + Cre: n = 46 dendritic segments, 627 individual mitochondria, mean length = 1.437 μm ± 0.040 (SEM), mean occupancy = 40.95% ± 1.94%; AMPK^FF/FF^ - Cre: n = 42 dendritic segments, 463 individual mitochondria, mean length = 1.418 μm ± 0.039 (SEM), mean occupancy = 39.24% ± 1.89%; AMPK^FF/FF^ + Cre: n = 36 dendritic segments, 381 individual mitochondria, mean length = 2.832 μm ± 0.116 (SEM), mean occupancy = 50.72% ± 2.36%, length increase = 97.08%, occupancy increase = 23.86%; Camkk2^-/-^: n = 46 dendritic segments, 449 individual mitochondria, mean length = 4.975 μm ± 0.233 (SEM), mean occupancy = 73.36% ± 2.03%, length increase = 367%, occupancy increase = 122%). A significant increase in mitochondrial length (**e**) and occupancy (**f**) is also seen in apical oblique dendrites when knocking out Camkk2, but only an increase in length is seen when knocking out AMPK. (WT: n = 28 dendritic segments, 184 individual mitochondria, mean length = 1.569 μm ± 0.104 (SEM), mean occupancy = 42.38% ± 2.98%; WT + Cre: n = 44 dendritic segments, 525 individual mitochondria, mean length = 1.791 μm ± 0.052 (SEM), mean occupancy = 48.50% ± 2.12%; AMPK^FF/FF^ - Cre: n = 45 dendritic segments, 440 individual mitochondria, mean length = 1.885 μm ± 0.075 (SEM), mean occupancy = 45.82% ± 2.29%; AMPK^FF/FF^ + Cre: n = 52 dendritic segments, 499 individual mitochondria, mean length = 2.645 μm ± 0.098 (SEM), mean occupancy = 47.79% ± 1.62%, length increase = 47.68%; Camkk2^-/-^: n = 29 dendritic segments, 288 individual mitochondria, mean length = 5.366 μm ± 0.346 (SEM), mean occupancy = 78.64% ± 2.07%, length increase = 242%, occupancy increase = 85.56%). Note that in Camkk2-null CA1 PNs we observe a significant increase in mitochondrial length (**h**) and occupancy (**i**) in the apical tuft compared to WT controls (WT: n = 33 dendritic segments, 226 individual mitochondria, mean length = 6.562 μm ± 0.437 (SEM), mean occupancy = 84.19% ± 1.44%; WT + Cre: n = 29 dendritic segments, 189 individual mitochondria, mean length = 5.206 μm ± 0.052 (SEM), mean occupancy = 79.40% ± 1.38%; AMPK^FF/FF^ - Cre: n = 38 dendritic segments, 229 individual mitochondria, mean length = 6.498 μm ± 0.368 (SEM), mean occupancy = 87.00% ± 1.67%; AMPK^FF/FF^ + Cre: n = 47 dendritic segments, 370 individual mitochondria, mean length = 5.723 μm ± 0.300 (SEM), mean occupancy = 80.65% ± 1.71%; Camkk2^-/-^: n = 35 dendritic segments, 199 individual mitochondria, mean length = 11.14 μm ± 0.758 (SEM), mean occupancy = 95.03% ± 1.73%, length increase = 69.77%, occupancy increase = 12.88%). p values are indicated in the figure following a one way ANOVA with Sidak’s multiple comparisons test. Data are shown as individual points on box plots with 25^th^, 50^th^ and 75^th^ percentiles indicated with whiskers indicating min and max values. Scale bar, 5 μm.

To test this in CA1 PNs in vivo, we performed CA1-targeted IUE in a constitutive *Camkk2* knockout mouse, as well as in a conditional *AMPKα1*^F/F^*α2*^F/F^ mouse (AMPK cDKO) and measured mitochondrial length and occupancy using a mitochondrial matrix-targeted fluorescent protein (mt-YFP, mt-DsRed) (Fig. 6). By electroporating Cre recombinase with a Cre-dependent reporter (FLEX-tdTomato, FLEX-mGreenLantern) in the AMPK cDKO, we could delete AMPK only from a subset of CA1 PNs. Using these approaches, we found that AMPK-null CA1 PNs display significantly increased mitochondria length and occupancy in basal (SO, Fig. 6a–c) and apical oblique (SR, Fig. 6d–f), but not in the apical tuft (SLM, Fig. 6g–i) dendritic compartments. Similarly, Camkk2-null CA1 PNs show a remarkably similar increase in mitochondrial size and occupancy in both SO and SR dendritic compartments (Fig. 6a–f). One interesting difference is that Camkk2-null CA1 PNs display a small but significant increase in mitochondria size and occupancy in the apical tuft compartment (SLM) not observed in the AMPK-null CA1 PNs. These results overall demonstrate that Camkk2 and AMPK are both required for the compartmentalized morphology of dendritic mitochondria observed in vivo and are specifically required for the small mitochondria size and dendritic occupancy in SO and SR dendritic compartments of CA1 PNs in vivo.

### Compartmentalized mitochondrial morphology in CA1 PNs requires Mtfr1l-dependent inhibition of Opa1

**Fig. 7:**
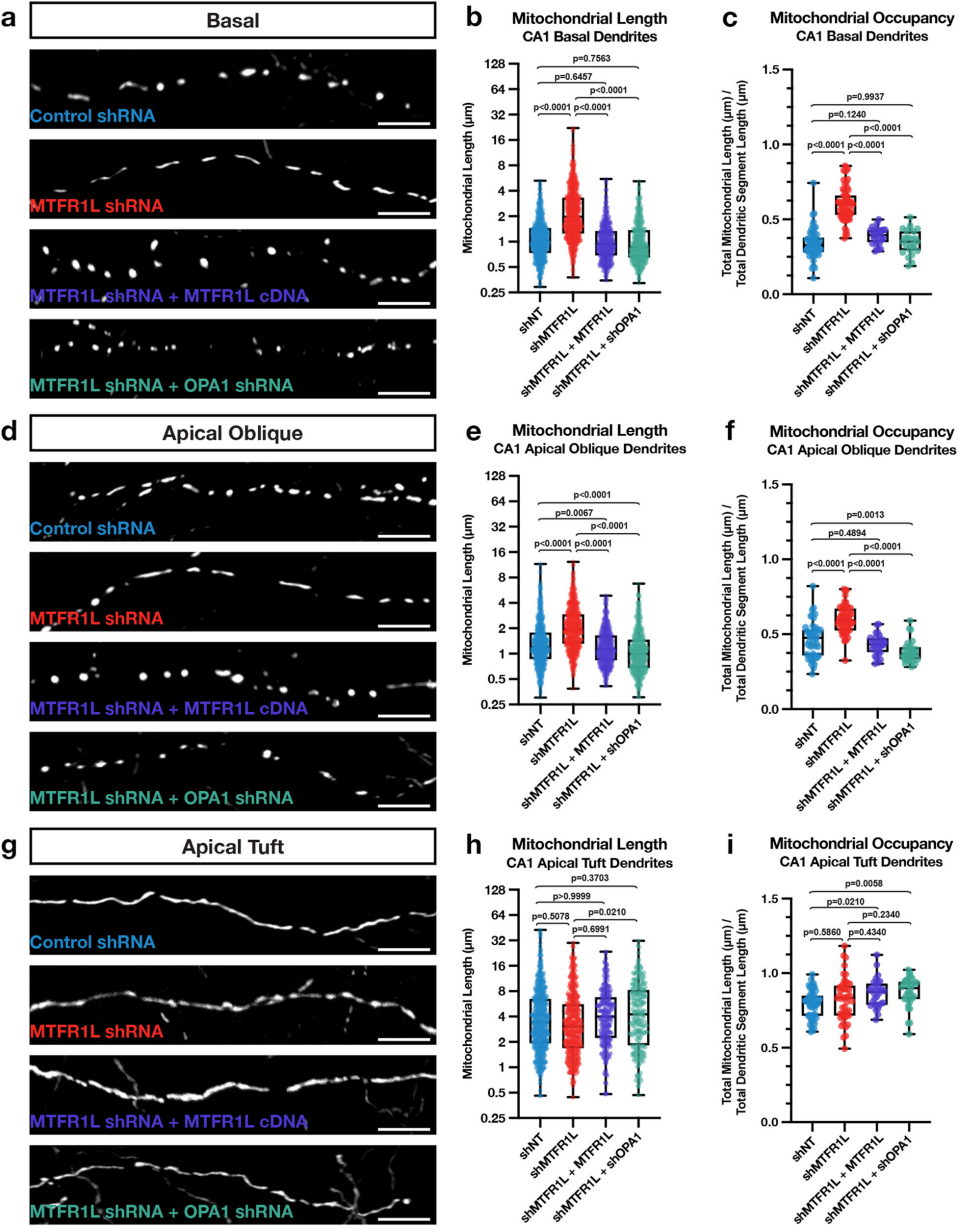
MTFR1L restricts hippocampal CA1 PN basal and apical oblique dendritic morphology through inhibition of Opa1. High magnification representative images of mitochondrial morphology within isolated secondary or tertiary hippocampal CA1 (**a**) basal, (**d**) apical oblique, and (**g**) apical tuft dendrites in which a mitochondrial matrix-targeted fluorescent protein (mt-YFP or mt-DsRed) and cell fill (tdTomato or mGreenLantern—not pictured) was *in utero* electroporated along with either a control shRNA (shNT), *Mtfr1l* shRNA (shMtfr1l), *Mtfr1l* shRNA with full-length hMTFR1L cDNA (shMtfr1l + hMTFR1L), or *Mtfr1l* shRNA and *Opa1* shRNA (shMtfr1l + shOpa1). Quantification of mitochondrial length and occupancy in the (**b**) and (**c**) basal, (**e**) and (**f**) apical oblique, and (**h**) and (**i**) apical tuft dendritic compartments reveals a significant increase in mitochondrial length (**b**) and occupancy (**c**) in basal dendrites when knocking down Mtfr1l, which is rescued when re-expressing full-length hMTFR1L or knocking down Opa1 (shNT: n = 51 dendritic segments, 670 individual mitochondria, mean length = 1.225 μm ± 0.028 (SEM), mean occupancy = 33.95% ± 1.46%; shMTFR1L: n = 60 dendritic segments, 773 individual mitochondria, mean length = 2.721 μm ± 0.085 (SEM), mean occupancy = 60.36% ± 1.46%, length increase = 122%, occupancy increase = 77.79%; shMtfr1l + hMTFR1L: n = 28 dendritic segments, 511 individual mitochondria, mean length = 1.113 μm ± 0.029 (SEM), mean occupancy = 39.10% ± 1.02%; shMtfr1l + shOpa1: n = 25 dendritic segments, 515 individual mitochondria, mean length = 1.126 μm ± 0.032 (SEM), mean occupancy = 35.08% ± 1.61%). A significant increase in mitochondrial length (**e**) and occupancy (**f**) is also seen in apical dendrites when knocking down Mtfr1l, which is similarly rescued when either expressing full-length hMTFR1L or additionally knocking down Opa1 (shNT: n = 48 dendritic segments, 669 individual mitochondria, mean length = 1.584 μm ± 0.059 (SEM), mean occupancy = 46.34% ± 1.72%; shMtfr1l: n = 54 dendritic segments, 683 individual mitochondria, mean length = 2.390 μm ± 0.061 (SEM), mean occupancy = 59.86% ± 1.29%, length increase = 50.88%, occupancy increase = 29.18%; shMtfr1l + hMTFR1L: n = 29 dendritic segments, 490 individual mitochondria, mean length = 1.352 μm ± 0.034 (SEM), mean occupancy = 42.92% ± 1.25%; shMtfr1l + shOpa1: n = 32 dendritic segments, 514 individual mitochondria, mean length = 1.248 μm ± 0.038 (SEM), mean occupancy = 38.28% ± 1.23%). No significant effect was observed following knockdown of Mtfr1l in the apical tuft dendrites in either mitochondrial length (**h**) or occupancy (**i**) compared to control neurons (shNT: n = 53 dendritic segments, 472 individual mitochondria, mean length = 5.177 μm ± 0.250 (SEM), mean occupancy = 79.14% ± 1.29%; shMtfr1l: n = 46 dendritic segments, 388 individual mitochondria, mean length = 4.665 μm ± 0.236 (SEM), mean occupancy = 82.35% ± 2.17%; shMtfr1l + hMTFR1L: n = 30 dendritic segments, 219 individual mitochondria, mean length = 5.186 μm ± 0.280 (SEM), mean occupancy = 86.65% ± 1.75%; shMtfr1l + shOpa1: n = 34 dendritic segments, 215 individual mitochondria, mean length = 5.872 μm ± 0.352 (SEM), mean occupancy = 87.36% ± 1.72%). p values are indicated in the figure following a one way ANOVA with Sidak’s multiple comparisons test. Data are shown as individual points on box plots with 25^th^, 50^th^ and 75^th^ percentiles indicated with whiskers indicating min and max values. Scale bar, 5 μm.

Previous results identified two direct effectors phosphorylated by AMPK mediating mitochondrial fragmentation: the Drp1-receptor Mff^24^ and the anti-fusion, Opa1-inhibiting protein Mitochondrial Fission Regulator 1-Like (Mtfr1l)^25^. We performed CA1-targeted IUE with cytoplasmic fluorescent proteins (tdTomato, mGreenLantern) and mitochondrial matrix-targeted fluorescent proteins (mt-YFP, mt-DsRed) in combination with either a control, non-targeting shRNA (shNT) or an shRNA targeting *Mtfr1l* (Fig. 7). The shRNA selected to knockdown Mtfr1l is validated in Fig. S5a, b. We found that knocking down Mtfr1l in CA1 PNs significantly elongates mitochondria and increases mitochondrial occupancy in the basal (SO; Fig. 7a–c) and apical oblique (SR; Fig. 7d–f) dendritic compartments, but not in apical tuft (SLM) dendrites (Fig. 7g–i). To confirm the specificity of our *Mtfr1l*-targeting shRNA, we co-expressed it together with a shRNA-resistant human cDNA expressing hMTFR1L. We found, as expected, that expression of hMTFR1L is sufficient to rescue mitochondrial length (Fig. 7b, e) and occupancy (Fig. 7c, f) back to levels observed in SO and SR dendritic compartments of control shRNA expressing CA1 PNs. Recent work demonstrated that Mtfr1l exerts its effects on mitochondria size by modulating the levels of the pro-fusion protein Opa1, and that functionally, Mtfr1l represents a novel class of ‘anti-fusion’ mitochondrial proteins because of its ability to oppose fusion and thereby enable Mff-dependent fission^25^. Based on these previously published results, we hypothesized that the mitochondrial elongation mediated by knocking down Mtfr1l should be rescued by simultaneously knocking down Opa1 in CA1 PNs. Therefore, we performed IUEs with shRNAs targeting both *Mtfr1l* and *Opa1* (see validation in Fig. S5e, f). Knocking down Opa1 completely rescues the effect of knocking down Mtfr1l and re-establishes short mitochondria and low occupancy in both basal and apical oblique dendrites to levels observed in control CA1 PNs (Fig. 7a–f). No significant effects were observed in dual knockdown of Mtfr1l and Opa1 in the apical tuft of the same neurons compared to knockdown of Mtfr1l alone or control shRNA expressing neurons (Fig. 7h, i). Overall, these results demonstrate that Mtfr1l is required for the compartmentalized morphology of mitochondria observed in the basal and apical oblique dendritic domains of CA1 PNs in vivo through the control of mitochondrial fusion and potentially inhibition of Opa1.

### Compartmentalized mitochondrial morphology in CA1 PNs requires Mff

**Fig. 8:**
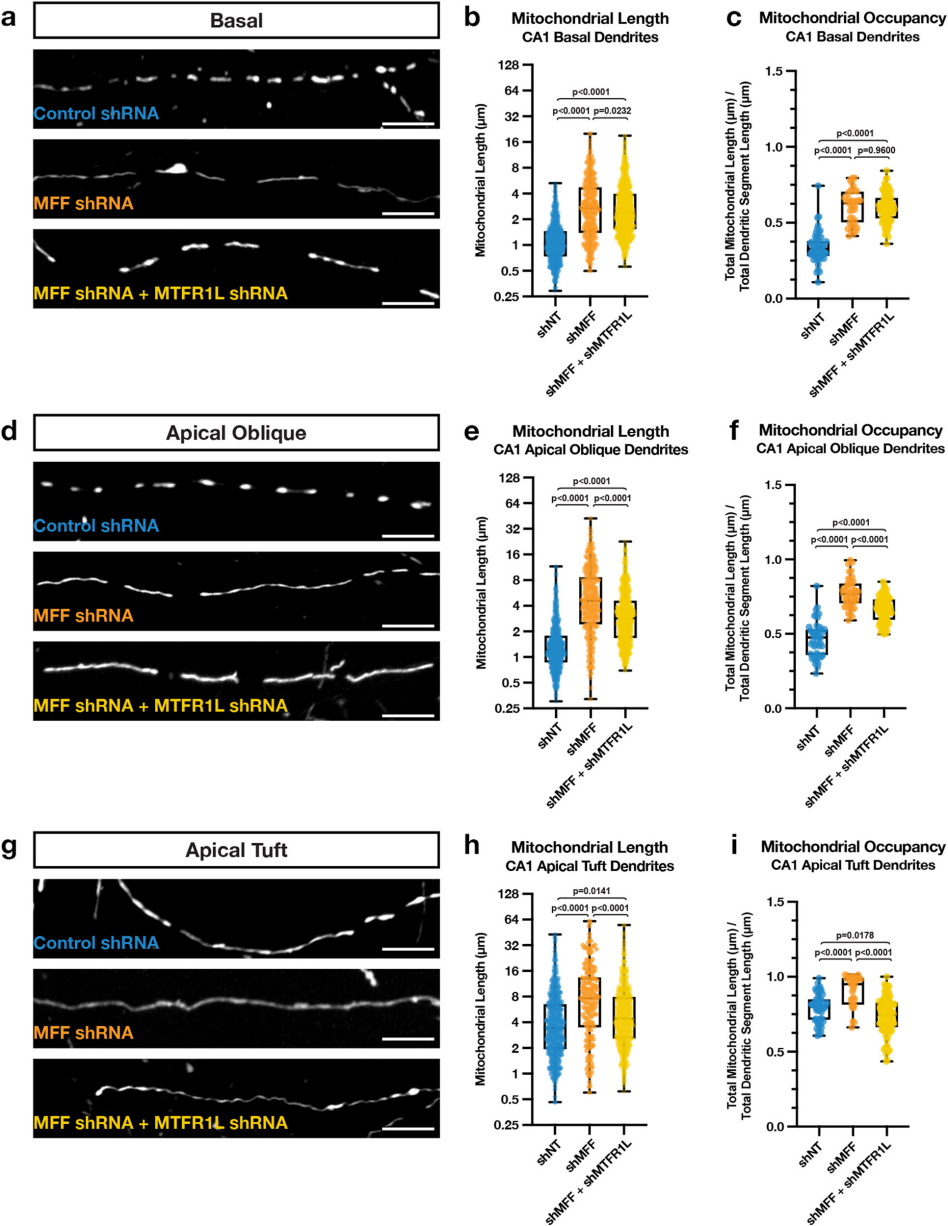
Mff restricts hippocampal CA1 PN basal, apical oblique, and apical tuft dendritic morphology. High magnification representative images of mitochondrial morphology within isolated secondary or tertiary hippocampal CA1 (**a**) basal, (**d**) apical oblique, and (**g**) apical tuft dendrites in which a mitochondrial matrix-targeted fluorescent protein (mt-YFP or mt-DsRed) and cell fill (tdTomato or mGreenLantern—not pictured) was *in utero* electroporated along with either a control shRNA (shNT), *Mff* shRNA (shMFF), or *Mff* and *Mtfr1l* shRNA (shMff + shMtfr1l). Quantification of mitochondrial length and occupancy in the (**b**, **c**) basal, (**e**, **f**) apical oblique, and (**h**, **i**) apical tuft dendritic compartments reveals a significant increase in mitochondrial length (**b**) and occupancy (**c**) in basal dendrites when knocking down Mff, which does not show an additive effect when also knocking down Mtfr1l (shNT: n = 51 dendritic segments, 670 individual mitochondria, mean length = 1.225 μm ± 0.028 (SEM), mean occupancy = 33.95% ± 1.46%; shMff: n = 36 dendritic segments, 380 individual mitochondria, mean length = 3.530 μm ± 0.152 (SEM), mean occupancy = 60.83% ± 1.86%, length increase = 188%, occupancy increase = 79.18%; shMff + shMtfr1l: n = 92 dendritic segments, 1004 individual mitochondria, mean length = 3.180 μm ± 0.078 (SEM), mean occupancy = 59.94% ± 0.99%, length increase = 160%, occupancy increase = 76.55%). A significant increase in mitochondrial length (**e**) and occupancy (**f**) is also seen in apical dendrites when knocking down Mff, but to a significantly less degree when knocking down both Mff and Mtfr1l (shNT: n = 48 dendritic segments, 669 individual mitochondria, mean length = 1.584 μm ± 0.059 (SEM), mean occupancy = 46.34% ± 1.72%; shMff: n = 44 dendritic segments, 371 individual mitochondria, mean length = 6.445 μm ± 0.300 (SEM), mean occupancy = 77.34% ± 1.46%, length increase = 307%, occupancy increase = 66.90%; shMff + shMtfr1l: n = 85 dendritic segments, 829 individual mitochondria, mean length = 3.728 μm ± 0.106 (SEM), mean occupancy = 66.63% ± 0.93%, length increase = 135%, occupancy increase = 43.79%). While there is a significant increase mitochondrial length (**h**) and occupancy (**i**) in the apical tuft when knocking down Mff alone, this increase largely goes away when knocking down both Mff and Mtfr1l (shNT: n = 53 dendritic segments, 472 individual mitochondria, mean length = 5.177 μm ± 0.250 (SEM), mean occupancy = 79.14% ± 1.29%; shMff: n = 31 dendritic segments, 199 individual mitochondria, mean length = 10.78 μm ± 0.757 (SEM), mean occupancy = 90.44% ± 1.86%, length increase = 109%, occupancy increase = 14.28%; shMff + shMtfr1l: n = 95 dendritic segments, 708 individual mitochondria, mean length = 6.326 μm ± 0.236 (SEM), mean occupancy = 73.79% ± 1.27%, length increase = 22.19%). p values are indicated in the figure following a one way ANOVA with Sidak’s multiple comparisons test. Data are shown as individual points on box plots with 25^th^, 50^th^ and 75^th^ percentiles indicated with whiskers indicating min and max values. Scale bar, 5 μm.

AMPK has also been shown to regulate mitochondrial morphology through direct phosphorylation of the OMM localized Drp1 receptor Mff^12,24^. To test if Mff is required for the compartmentalized morphology of mitochondria in dendrites of CA1 PNs, we repeated the same IUE experiments using an shRNA targeting *Mff* (shMff) previously characterized^7^, and we re-validated the shRNA efficiently knocks down all isoforms of Mff (Fig. S5c, d). Knocking down Mff in CA1 PNs throughout development using IUE, we observe a significant increase in mitochondrial length and occupancy in all three dendritic compartments (Fig. 8a–i). Since knockdown of either Mtfr1l or Mff leads to similar phenotypes on mitochondria morphology, especially in basal and apical oblique dendritic domains of CA1 PNs, we tested if these two proteins have redundant or only partially overlapping functions in regulating dendritic mitochondrial morphology in these neurons. Interestingly, we found that knocking down both Mtfr1l and Mff does not lead to additive effects compared to knocking down Mff or Mtfr1l separately (Fig. 8a–f), strongly arguing that they play redundant functions or that they regulate their mutual abundance at OMM. In fact, we discovered that knockdown of either Mff or Mtfr1l significantly decreased protein abundance of the other; however, knockdown of Mff had a stronger impact on Mtfr1l expression (Fig. S5g, h). These results explain the observations that Mff knockdown has a greater effect on mitochondrial compartmentalization of CA1 PNs in vivo, and do not seem to act synergistically in their dendrites.

### Camkk2 regulates morphological compartmentalization of dendritic mitochondria in CA1 PNs through AMPK-dependent Mtfr1l phosphorylation

**Fig. 9:**
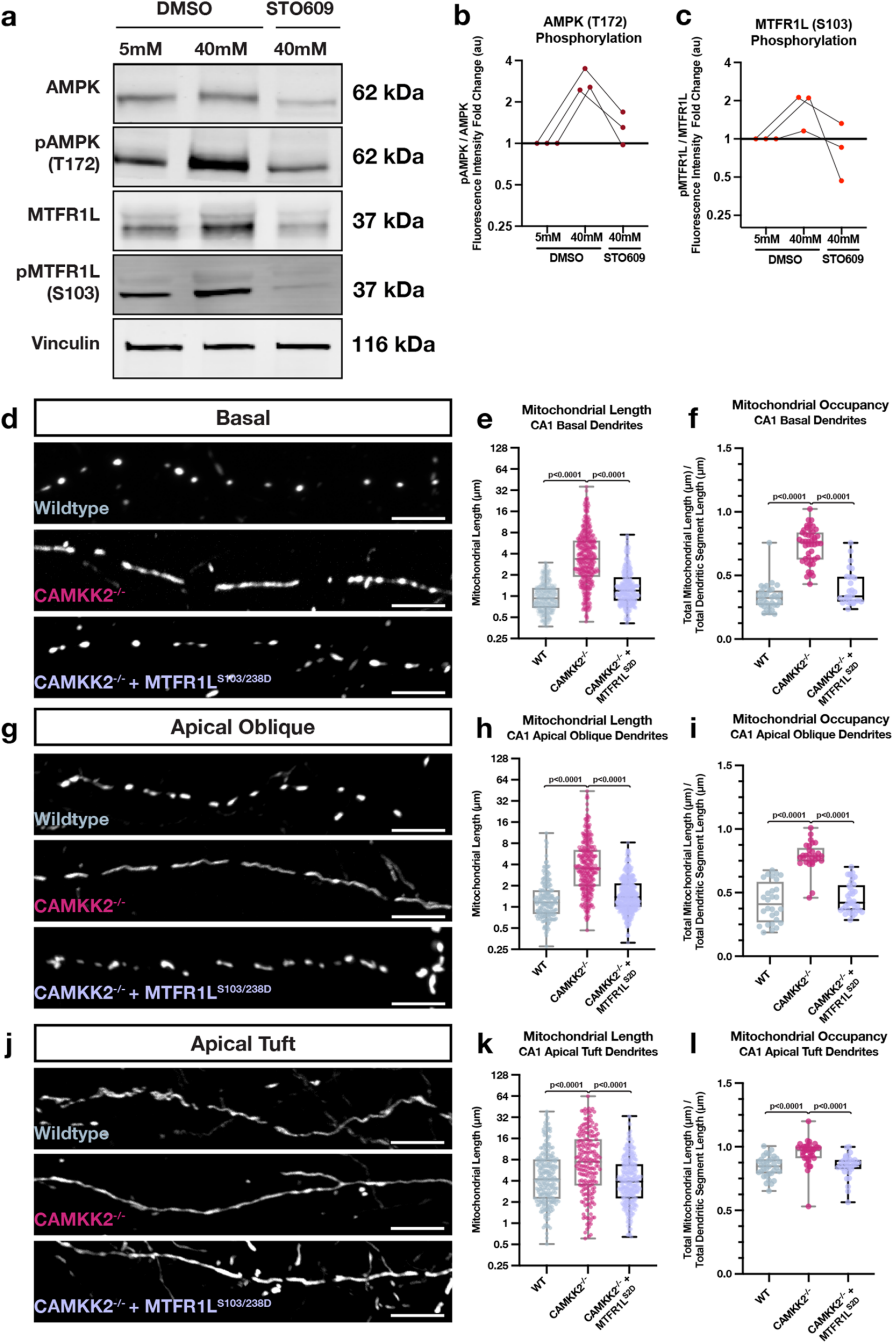
Activity-dependent and Camkk2-dependent phosphorylation of MTFR1L by AMPK mediates compartmentalized mitochondria morphology in dendrites of CA1 PNs in vivo. **a** Western blots of whole cell lysates from mouse hippocampal neurons maintained in culture for 18-21DIV and treated for 15 min with physiological (5 mM) extracelllular potassium chloride (KCl) (first column) or high KCl (40 mM) inducing membrane depolarization (columns 2 & 3) in the presence (column 3) or absence (columns 1 & 2) of the Camkk2 inhibitor STO609. These results demonstrate that phosphorylation of AMPKα catalytic subunit on T172 is increased by neuronal depolarization which is blocked by STO609. In turn, AMPK phosphorylation of its substrate MTFR1L on S103^25^ is increased by depolarization which is Camkk2-dependent since it is blocked by STO609. **b** Quantification of western blots with fold change of fluorescence intensity of pAMPK normalized to total AMPK plotted for each condition relative to 5 mM KCL treatment. **c** Quantification of western blots with fold change of fluorescence intensity of pMTFR1L normalized to total MTFR1L plotted for each condition relative to 5 mM KCL treatment. (d-l) Rescue experiments showing that phosphomimetic form of Mtfr1l (Mtfr1l^S2D^) mimicking phosphorylation by AMPK^25^ is sufficient to rescue compartmentalized mitochondria morphology in basal (**d**–**f**), apical oblique (**g**–**i**) and apical tufts (**j**–**l**) dendrites of CA1 PNs in vivo. CA1 PNs from wild-type (WT) or *Camkk2*^-/-^ constitutive knockout mice were IUE with the same mitochondrial markers/cell fills as in Fig. 3 (WT and Camkk2^-/-^) and a plasmid cDNA expressing phosphomimetic mutant on the two serine residues phosphorylated by AMPK (S103D and S238D) of Mtfr1l (Mtfr1l^S2D^)^25^. Data and quantifications from WT and Camkk2^-/-^ are the same as in Fig. 6. Basal Dendrites (WT and Camkk2^-/-^: See Fig. 6; Camkk2^-/-^ + Mtfr1l^S2D^: n = 28 dendritic segments, 375 individual mitochondria, mean length = 1.583 μm ± 0.061 (SEM), mean occupancy = 39.99% ± 2.12%). Apical Oblique Dendrites (WT and Camkk2^-/-^: See Fig. 3; Camkk2^-/-^ + Mtfr1l^S2D^: n = 37 dendritic segments, 459 individual mitochondria, mean length = 1.743 μm ± 0.053 (SEM), mean occupancy = 46.19% ± 2.22%). Apical Tuft Dendrites (WT and Camkk2^-/-^: See Fig. 3; Camkk2^-/-^ + Mtfr1l^S2D^: n = 30 dendritic segments, 315 individual mitochondria, mean length = 5.310 μm ± 0.262 (SEM), mean occupancy = 84.00% ± 1.31%). Experiments in a were replicated 3 times. For (**e**), **f**, (**h**), (**i**), (**k**), and (**l**), p values are indicated in the figure following a one way ANOVA with Sidak’s multiple comparisons test. For (**b**) and (**c**), individual points from independent experiments are shown with connected lines. For (**e**), (**f**), (**h**), (**i**), (**k**), and (**l**), data are shown as individual points on box plots with 25^th^, 50^th^ and 75^th^ percentiles indicated with whiskers indicating min and max values. Scale bars, 5 μm.

We next tested whether the kinase dyad Camkk2-AMPK regulates the compartment-specific morphology of mitochondria in CA1 PNs through phosphorylation of Mtfr1l. We used two experimental approaches to test this. First, we performed biochemical analysis in mouse hippocampal neurons in vitro to test if Mtfr1l phosphorylation by AMPK is regulated by neuronal activity, and, whether activity-dependent regulation of Mtfr1l phosphorylation is Camkk2-dependent. To this end, we treated hippocampal neurons maintained in dissociated cultures for 3 weeks to a high potassium chloride (KCl) concentration (switch from 5 to 40 mM) for a short time period (15 min; Fig. S5k, l) to induce strong membrane depolarization and effectively open VGCCs leading to increased intracellular Ca2+ in hippocampal neurons^42^. As shown previously^26,41,43^, western blot analysis revealed that neuronal depolarization induces increased AMPK phosphorylation on T172 in a Camkk2-dependent manner since it was blocked by a Camkk2-specific inhibitor STO609 (Fig. 9a). In the same lysates, KCl-mediated neuronal depolarization induced an increase in Mtfr1l phosphorylation on S103, one of the two serine residues phosphorylated by AMPK^25^ that was also blocked by STO609 (Fig. 9a–c). These results demonstrate that, in hippocampal neurons, Mtfr1l is phosphorylated by AMPK in an activity- and Camkk2-dependent manner.

**Fig. 10:**
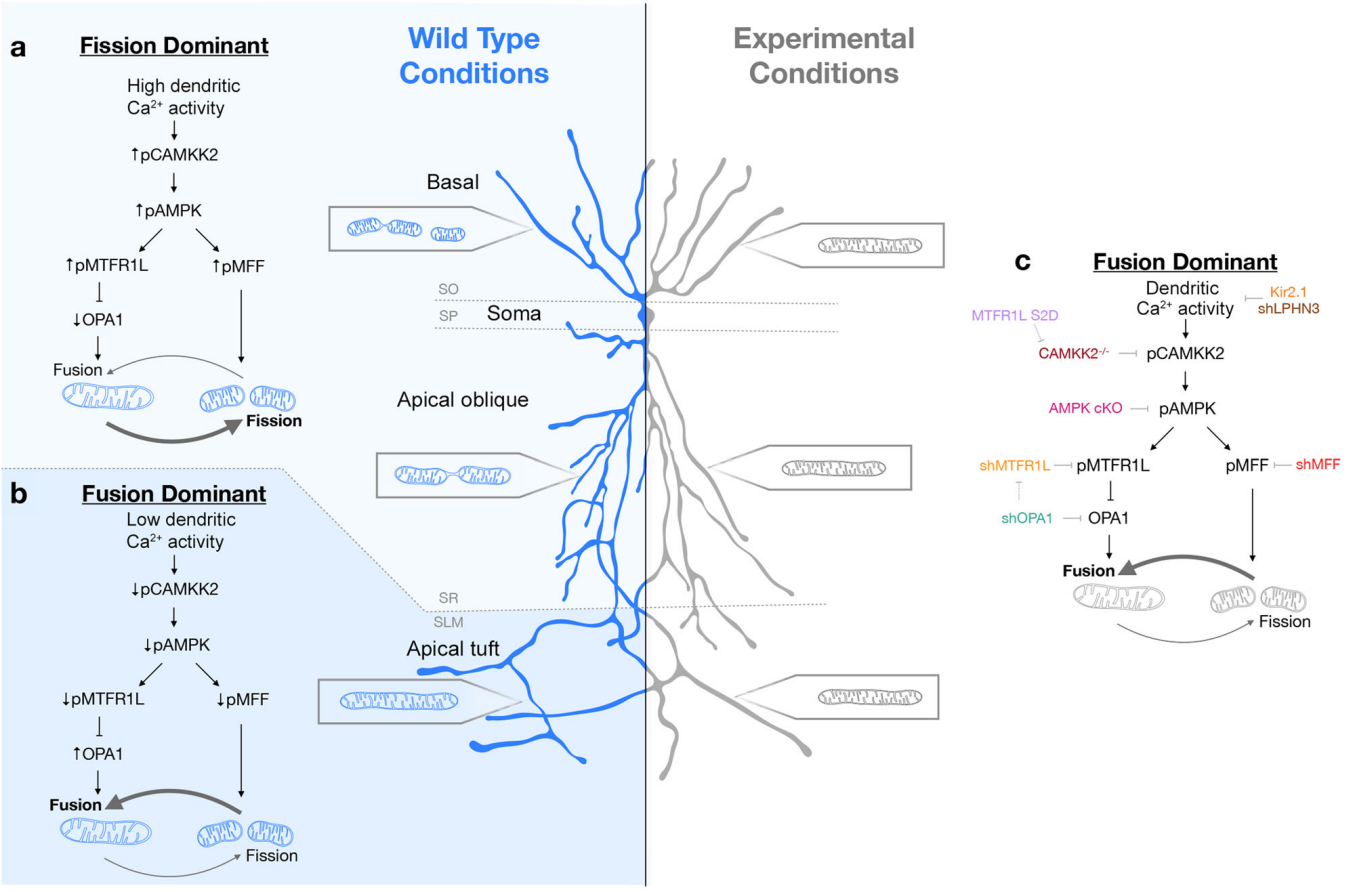
Summary of the main findings. **a**, **b** In wild-type mouse CA1 PNs, dendritic mitochondria display a striking degree of compartmentalized morphology, being long and fused in the apical tufts (SLM) with progressive fragmentation and occupancy of a smaller volume of the dendritic segments in SR and SO respectively. **c** We demonstrate using loss-of-function as well as rescue experiments that this compartmentalization of dendritic mitochondria morphology in CA1 PNs in vivo requires (1) neuronal activity (blocked by neuronal hyperpolarization following over-expression of Kir2.1) or by reducing the number of presynaptic inputs from CA3 to SR and SO dendrites by ~50% (shRNA Lphn3) in vivo, (2) requires activity-dependent activation of AMPK mediated by Camkk2 and (3) requires the AMPK-dependent phosphorylation of the pro-fission Drp1 receptor Mff and the anti-fusion protein Mtfr1l though its ability to suppress the pro-fusion Opa1 protein. These results demonstrate that mitochondrial fusion dominates over fission in apical tuft dendrites (SLM) and that activity-dependent mitochondrial fission dominates over fusion in both SO and SR dendritic compartments. See Discussion for details.

Next, we tested whether Camkk2-dependent Mtfr1l phosphorylation by AMPK is required for the compartmentalized mitochondrial morphology characterizing CA1 PNs in vivo. Previous work demonstrated that AMPK phosphorylates two conserved serine residues on Mtfr1l (positions S103 and S238), and that a phosphomimetic form of Mtfr1l^S103D/238D^ (hereafter referred to as Mtfr1l^S2D^) is sufficient to rescue mitochondria morphology in AMPK-null cells^25^. Therefore, we reasoned that if Camkk2 regulates the dendritic compartmentalization of mitochondrial morphology in CA1 PNs by AMPK-dependent phosphorylation of Mtfr1l, expression of phosphomimetic Mtfr1l^S2D^ should rescue the mitochondrial morphology defects observed in Camkk2-null CA1 PNs in vivo. Indeed, we found that expression of Mtfr1l^S2D^ in Camkk2-null CA1 PNs rescues the morphology of dendritic mitochondria both in SR and SO compartments back to levels observed in wild-type mice (Fig. 9d–l). These results demonstrate that Camkk2 mediates compartmentalization of mitochondrial morphology through AMPK-dependent phosphorylation of Mtfr1l in SO and SR dendritic compartments where fission dominates over fusion (Fig. 10).

## Discussion

In this study, we identified molecular and cellular effectors that enable synaptic activity to shape the compartment-specific morphology of mitochondria in neuronal dendrites. In the apical tufts (SLM) of CA1 pyramidal neurons, mitochondria are elongated, tubular, and fill a high fraction of the dendritic volume; in contrast, the mitochondria found in more proximal dendritic compartments are short and occupy a significantly smaller fraction of dendritic volume. This striking compartmentalization of mitochondria morphology corresponds to dendritic domains receiving different presynaptic inputs, from the entorhinal cortex in SLM and from CA2/3 in SR and SO. Collectively, our results (summarized in Fig. 10) demonstrate that the restricted mitochondrial morphology observed in the SR and SO dendritic compartments (compared to SLM) is shaped by synaptic activity via Camkk2-dependent AMPK phosphorylation of the pro-fission Drp1 receptor Mff^12,24^ and phosphorylation of the anti-fusion Mtfr1l^25^, a protein antagonizing Opa1.

Our data strongly supports a model where low, activity-dependent, dendritic Ca^2+^ dynamics inhibits the engagement of the mitochondrial fission machinery, as the elongated mitochondria morphology observed in the apical tufts (SLM) of CA1 PNs is present across the entire dendritic arbor in experimental conditions where we reduce the number of synaptic inputs or neuronal activity (Fig. 10). In support of this concept, experimentally reducing neuronal activity through expression of Kir2.1 or cell-autonomously reducing by ~50% the number of CA3 inputs received by individual CA1 PNs (Fig. 3), is sufficient to induce tubular and fused mitochondria morphology in the SO and SR dendritic compartments in vivo (Fig. 3). Therefore, our data suggests that presynaptic activity and cytoplasmic Ca^2+^ dynamics in basal and apical oblique dendrites of CA1 PNs drives the Camkk2-dependent activity which triggers high levels of AMPK kinase activity and phosphorylation of the anti-fusion effector Mtfr1l (Fig. 7^25^) and pro-fission Mff (Fig. 8^12,24^). In the apical tuft of CA1 PNs, we demonstrated lower amplitude and frequency of cytoplasmic Ca^2+^ transients compared to more proximal apical oblique (SR) dendritic compartment in vivo (Fig. 2). Combined with our biochemical experiments demonstrating that AMPK through phosphorylation of its direct substrate Mtfr1l^25^ are regulated by neuronal depolarization in a Camkk2-dependent manner, our results strongly argue that (1) low levels of Ca^2+^-dependent Camkk2/AMPK activity and therefore low levels of Mff and Mtfr1l activity lead to fusion dominating over fission in apical tufts of CA1PNs, whereas (2) in more proximal dendritic compartments of the same neurons, high levels of cytoplasmic Ca^2+^ transients lead to high levels of Camkk2-AMPK-dependent Mff-dependent fission and Mtfr1l-dependent anti-fusion to dominate over fusion (Fig. 10). A recent study, conducted using mostly in vitro approaches, confirmed our previous^12,26^ and our current results and found that the Camkk2-AMPK kinase dyad is regulated by neuronal activity and that its inhibition led to dysregulation of mitochondrial homeostasis and reduction in dendritic complexity in hippocampal neurons^44^. In the future, it will be important to determine if the rates of mitochondrial fusion to fission are unbalanced in distinct dendritic compartments of CA1 hippocampal neurons in vivo thus allowing for mitochondrial fusion to dominate, or if other mitochondrial processes (e.g. trafficking, biogenesis or mitophagy) might also play a role in the development of mitochondrial morphological compartmentalization in neuronal dendrites.

What could be the functional consequence of this striking compartmentalized mitochondrial morphology in dendrites of CA1 PNs? As mitochondria buffer a significant fraction of the Ca^2+^ released from the ER in dendrites of CA1 PNs^45^, one potential consequence of the reduced mitochondrial volume in SR/SO dendrites could be that mitochondria in these compartments have a reduced capacity to buffer Ca^2+^, therefore representing a positive-feedback loop allowing more cytoplasmic Ca^2+^ to accumulate in these compartments upon synaptic activity, further increasing local Camkk2/AMPK activity in SR and SO dendritic domains. In the apical tufts of the same neurons, lower synaptic activity (spine density is ~40% lower in SLM than in SO/SR dendrites^12,26,30,46^), might trigger lower amplitude/frequency of Ca^2+^ transients and therefore lower levels of Camkk2/AMPK activity allowing mitochondrial fusion to dominate over Mff-dependent fission.

The molecular effectors identified in the present study will enable future investigations of the functional impact of this striking degree of compartmentalization of mitochondrial morphology on dendritic integration properties, synaptic plasticity, and circuit properties of CA1 PNs in vivo^45^. Mitochondria structure and function have been proposed to play multiple roles beyond ATP generation including control of local protein synthesis^47^ and the emergence of synaptic and circuit properties underlying normal brain function^45,48–52^ or the pathophysiological mechanisms underlying of various neurodegenerative disorders^53^. Future investigations will have to test (1) the functional consequences of the activity-dependent mechanisms regulating compartmentalized mitochondrial morphology uncovered in the present study for CA1 PNs and (2) if this striking degree of compartmentalization of mitochondrial morphology applies to the dendrites of other neuronal subtypes in the central nervous system. Activity-dependent gene transcription has been studied extensively for its role in regulating neuronal morphogenesis and connectivity during development and synaptic plasticity in adult circuits^54^ but to our knowledge has never been involved in regulating mitochondria (or any other organelle) biogenesis, structure or function in neurons. Interestingly, in developing astrocytes, mGLuR5 signaling controls their maturation through regulation of PGC1a-dependent mitochondria biogenesis^55^. Future investigations will determine if, in neurons, the activity-dependent signaling pathway identified here, operating in a spatially-restricted manner in the dendrites of CA1 PNs in vivo, also involves transcriptional and/or translational regulation.

## Methods

### Animals

All experiments involving mice were done according to protocols approved by the Institutional Animal Care and Use Committee (IACUC) at Columbia University, Oklahoma Medical Research Foundation (OMRF) and in accordance with National Institutes of Health guidelines. Animal health and welfare were supervised by a designated veterinarian. All mice were maintained in Columbia University or OMRF animal facilities that comply with all appropriate standards of care, including cage conditions, space per animal, temperature, humidity, food, water, and 12-hour light/dark cycles.

Timed-pregnant hybrid F1 control females were obtained by mating inbred *129/SvJ* females (Charles River) and *C57BL/6* *J* males in house. Homozygous double conditional knockout (cKO) lines AMPKα1^F/F^α2^F/F^ ^56^ were provided by Dr Benoit Viollet (INSERM, Institut Cochin- Paris, France) and homozygous *Camkk2*^-/-^ ^57^ were kindly obtained from Dr. Talal Chatila (Harvard Medical School, Boston). Both AMPKα1^F/F^α2^F/F^ double cKO and *Camkk2*^-/-^ timed-pregnant females were obtained by mating homozygous males with females of the same genotype. CD-1 IGS mice (Strain Code: 022) were purchased from Charles River Laboratories.

### Cell and Tissue Lysis and Western Blotting

Human Embryonic Kidney 293 T/17 (HEK293T) cells were purchased from ATCC (CRL-11268). 1 × 10^5^ HEK cells were resuspended in media (DMEM, Gibco) with penicillin/streptomycin (0.5×; Gibco) and FBS (Sigma) and seeded in 6 well plates (Corning). Transfection with plasmid DNA (1 mg/mL) using jetPRIME® reagent (Polyplus) according to manufacturer protocol was performed 24 hours after seeding. 72 hours following transfection, cells were carefully washed with 1xPBS (Gibco) then collected into RIPA buffer with protease inhibitor cocktail.

Aliquots of the collected samples were separated by SDS-PAGE and then transferred to a polyvinylidene difluoride (PVDF) membrane (Amersham). After transfer, the membrane was washed 3X in Tris Buffer Saline (10 mM Tris-HCl pH 7.4, 150 mM NaCl) with 0.1% of Tween 20 (T-TBS), blocked for 1 hr at room temperature in Odyssey Blocking Buffer (TBS, LI-COR), followed by 4 °C overnight incubation with the appropriate primary antibody in the above buffer. The following day, the membrane was washed 3X in T-TBS, incubated at room temperature for 1 hr with IRDye secondary antibodies (LI-COR) at 1:10,000 dilution in Odyssey Blocking Buffer (TBS), followed by 3X T-TBS washes. Visualization was performed by quantitative fluorescence using an Odyssey CLx imager (LI-COR). Signal intensity was quantified using Image Studio software (LI-COR). Primary antibody used for Western blotting was mouse anti-LPHN3 (R&D Systems MAB5916, 1:1000) and rabbit anti-GAPDH (CST 2118, 1:5000). Secondary antibody used for Western blotting was goat anti-rabbit IRDye680RD (LICOR #926-68071) and goat anti-mouse IRDye800CW (LICOR #926-32210). Total protein was assessed using the Revert 700 Total protein stain (LI-COR 926-11010).

### Activity assay

#### Cell culture and lysis

Embryos were harvested at E15.5 and chilled in Hank’s Balanced Salt Solution (HBSS, Thermo Fisher Scientific) while medial portion of the dorsal telencephalon (presumptive hippocampi) were dissected out. Isolated hippocampi were incubated in papain for 15 minutes at 37 °C. After three washes of HBSS, hippocampi were manually dissociated with a pipette into Neurobasal (Thermo Fisher Scientific) supplemented with FBS (Gemini Bio-Products), GlutaMAX (Thermo Fisher Scientific), penicillin/streptomycin (Thermo Fisher Scientific), B27 (Fisher Scientific), and N2 (Thermo Fisher Scientific). Dissociated cells were plated into the wells of 6 well plates (Corning) (7.5 × 10^5^ cells/well) that were previously coated in Poly-D-Lysine (Thermo Fisher Scientific) and incubated at 37 °C with 5% CO2. Cells were fed every 3-4 days by removing 0.75 mL of media and replenished with 1 mL of fresh Neurobasal containing GlutaMAX, B27 and N2 only. Neurons were cultured to 21 days in vitro (DIV) and then treated with either DMSO (2.5 µM, Santa Cruz Biotechnology) or STO609 (2.5 µM, Sigma-Aldrich) for 2.5 hours. Neurobasal media was then switched out for 5 K Tyrode Solution containing TTX (1 µM, Hello Bio) to block sodium channels, NMDA antagonist AP5 (10 µM, Hello Bio), and AMPA antagonist NBQX (50 µM, Hello Bio) for 30 minutes to dampen basal levels of neuronal activity. Media was then switched out for either a second incubation in 5 K (5 mM KCl) or 40 K (40 mM KCl) in Tyrode Solution for 15 minutes. Drug treatments were maintained throughout every incubation step. Tyrode Solution was removed, and cells were lysed on ice with 100 µl of N-PER (Thermo Fisher Scientific) containing phosphatase inhibitors (Sigma Aldrich), protease inhibitor (Sigma Aldrich), and benzonase (EMD Millipore). Lysates were then denatured in 4x Laemmli Buffer (BioRad) and 2-Mercaptoethanol (BioRad) at 95 °C for 5 minutes.

#### Western Blotting

Equal amounts of lysates were loaded onto Mini-Protean TGX (4-20%) SDS-PAGE gels (BioRad). All blots except phospho-MTFR1L were transferred to nitroceullose membranes (BioRad) with a Trans-Blot Turbo and blocked for one hour in Intercept Buffer (Li-COR). Membranes were then incubated in their respective primary antibodies in blocking buffer at 4 °C overnight. Blots probing for phospho-MTFR1L we transferred to polyvinylidene difluoride membranes (PVDF, Immobilion) and blocked in 5% fat-free dry milk in TBS-T for 1 hour before primary antibody incubation for four days at 4 °C. Membranes were incubated in Li-Cor fluorescence-coupled secondary antibodies for 1 hour at room temperature prior to visualization with a Li-Cor Odyssey Blot Imager.

#### Antibodies

rabbit anti-AMPKα (Cell Signaling, #2532 S, 1:2000); rabbit anti-phospho-AMPKα (Cell Signaling, #2535 S, 1:2000); rabbit anti-MFF (Proteintech #17090-1-AP, 1:1000); rabbit anti-MTFR1L (Atlas Antibodies #HPA027124, 1:2000); rabbit anti-phospho-MTFR1L (S103, 1:500; characterized in^25^); goat anti-Rabbit IRDye800CW (LI-COR #926-32211, 1:10,000).

### Lentiviral particle production and transduction

Lentiviral particles were generated as described before (https://www.addgene.org/protocols/lentivirus-production/). Briefly, freshly thawed HEK293T cells (ATCC, CRL-3216) were seeded in a 10 cm dish containing DMEM (GIBCO, 12430-054) supplemented with 10% FBS (Thermo Scientific, 16141002), 1% GlutaMax (GIBCO, 35050061), 1% Sodium Pyruvate (GIBCO, 11360070), and 1% Pen / Strep (Life Technologies, 15140-122), and grown at 37 °C, 5% CO_2_ for ~ 48 hours. Once confluent, cells were detached from the dish by trypsinization (GIBCO, 25200072), split into 15 cm dishes, and grown to ~80 % confluence. Before transfection, cultures were treated with 25 μM chloroquine diphosphate for 5 h, supplemented in fresh growth media. For one 15 cm dish, the transfection reaction was assembled in two separate tubes, each containing 1.5 ml serum-free DMEM. The plasmid mixture, composed of pMD2.G (Addgene, #12259), psPAX2 (Addgene, #12260), and a designated transfer vector, was added to the first tube, and the transfection reagent Polyethylenimine (PEI) to the second tube. Both halves were mixed and incubated at RT for 20 min, time after which the reaction was added dropwise to the dish. Cultures were incubated with the transfection reaction for 24 h and then replaced by fresh growth media. Media containing lentiviral particles was collected 72 h after transfection and spun down for 5 min at 2000 g to pellet cell debris. The supernatant was filtered through a 0.45 μm pore, aliquoted in 1.5 ml Eppendorf tubes, and centrifuged for 2 h at 16000 g at 4 °C. The supernatant was discarded, and dry viral pellets were stored at −80 °C until use. For neuronal transduction, viral pellets were resuspended in serum-free Neurobasal medium (GIBCO, 21103049), added to neuronal cultures, and incubated for 5-7 days before sample collection.

### Single cell electroporation (SCE) of CA1 PNs

SCEs were performed as previously described in detail^45,58^. Briefly, DNA plasmid constructs were diluted to 50 ng/µL and electroporated into single dorsal hippocampal CA1-area pyramidal neurons, guided by 2-photon microscopy at 920 nm excitation. Successfully electroporated cells were imaged 48-72 h post SCE.

### In utero electroporation (IUE) targeting CA1 PNs

In utero electroporation targeting the dorsal hippocampus was performed using a triple-electrode setup as previously described^46,59,60^ in E15.5 mouse embryos in order to target the dividing progenitors generating CA1 pyramidal neurons. Briefly, endotoxin-free plasmids were injected into the lateral ventricle (1 μg/μL) followed by 5 pulses at 45 V (50 ms duration, 500 ms inter-pulse interval) using two anodes (positively charged) laterally on either side of the head and one cathode (negatively charged) rostrally at a 0° angle to the horizontal plane. Mice were perfused transcardially with ice cold PFA (2-4%) and glutaraldehyde (0.075%) at P21, post-fixed overnight in the same solution at 4 C, and then sectioned at 110-125 μm on a vibratome (Leica). Sections were mounted with Fluoromount-G aqueous mounting medium (ThermoFisher Scientific) and imaged on a Nikon Ti-E A1R laser-scanning confocal microscope using a 40x or 60x NA1.49 objective, or a Zeiss LSM 880 confocal microscope controlled by Zeiss Black software. Imaging required two lasers 488 nm and 561 nm together with Zeiss objectives 20x (1.2NA) with 2x zoom, or 100x oil (1.25NA) with 3x zoom. Neuronal processes were visualized by Z-stacking that was later processed into Maximum Intensity Projection (MIP). MIP 2D images of the cell fill were used to choose branches blinded to mitochondria phenotype. Mitochondria length and occupancy were then traced using the segmentation tools in NIS Elements software (Nikon) or Fiji (Image J) and measured along the axis in line with the length of the dendrite. Occupancy is the sum of all mitochondrial length in a given branch divided by branch length.

For acute induction of ERT2-Cre-ERT, mice were dosed with 4-OHT at P21 and P22 via intraperitoneal (IP) injection. 4-OHT was stored at −80°C in Cremophor (Sigma #238470) at 10 mg/ml. For delivery, aliquots were diluted 1:10 in PBS. ~20 μg/g of body weight was injected per mouse on both days. Mice were perfused with fixatives, as described above, on P23.

### Primary neuronal culture

Following *in utero* electroporation at E15.5, mouse embryos were collected at E18.5 and medial parts of the dorsal telencephalon corresponding to the nascent hippocampus were dissected in Hank’s Balanced Salt Solution (HBSS) supplemented with HEPES (10 mM, pH 7.4), and incubated in HBSS containing papain (Worthington; 14 U/mL) and DNase I (100 μg/mL) for 15 min at 37 °C with a gentle flick between incubation. Samples were washed with HBSS three times and dissociated by pipetting on the fourth wash. Cells were counted using Countess™ (Invitrogen) and cell suspension was plated on poly-D-lysine (1 mg/mL, Sigma)-coated glass bottom dishes (MatTek) or poly-D-lysine/laminin coated coverslips (BD bioscience) in Neurobasal media (Gibco) containing FBS (2.5%) (Sigma), B27 (1 X) (Gibco), and Glutamax (1 X) (Gibco). After 7 days, media was changed with supplemented Neurobasal media without FBS, and 1/3 of the media replaced every 3 days.

### Measurement of activity dependent fission and fusion rates

At E15.5, CD-1 embryos were ex utero electroporated with pCAG mt-paGFP:p2a:mt-mScarlet (0.5 µg/µl) and dissociated cortical neuron cultures were prepared as above described above for hippocampal neurons with the exception that cortex was used. At 13DIV, dimethyl sulfoxide (DMSO, Sigma, 1:1000) or picrotoxin (PTX, Tocris, 50 µM) or picrotoxin and STO-609-acetic acid (PTX + STO609, Sigma, 1.25 µM) was added to the culture medium overnight for 12-16 hours. At 14DIV, treated neurons were live imaged on a Nikon Ti2e equipped with a Okolab stage top incubator, Hamamatsu Fire camera, Lumencor AURA light engine (365 nm, 488 nm, 561 nm, 647 nm), and Optimicroscan XY galvo scanning unit with a 405 nm stimulation laser. Briefly, a 8 nm by 8 nm square ROI was selected on a dendrite of interest and the stimulation laser was used to deliver a single low pulse of 405 nm light (50 µs per pixel at 1% laser power) to photoactivate the mitochondria localized paGFP. The dendrite with photoactivated GFP was then imaged every 10 seconds for 15 minutes to capture fission and fusion events of the photoactivated mitochondria under each intervention. Photoactivated mitochondria were manually tracked and fission and fusion events counted in NIS Elements AR software.

### Fixation for primary neuron culture

Culture dishes were fixed with 2% PFA (PFA Alfa Aesar)/0.075% GA (Electron Microscopy Science, EMS) in 1x PBS (Sigma) for 10 minutes. Dishes were washed three times following fixation with 1x PBS (Sigma) for 10 minutes.

### Immunohistochemistry

Following fixation and washing, brains were embedded in 3% low melt agarose (RPI, A20070) in 1x PBS. Brains in agarose cubes were sectioned using a vibratome (Leica VT1200) to 120 μm thick sections. Sections/coverslips were then incubated with primary antibodies (chicken anti-GFP Aves Lab 1:4000, rabbit anti-dsRed Abcam 1:4000) that were diluted in the Blocking buffer (1%BSA, 0.2%TritonX-100, 5%NGS in PBS) at 4 °C for 48 h. Subsequently sections were washed 6 times for 10 min in PBS and incubated with secondary antibodies (Alexa conjugated goat anti-chicken488 and goat anti-rabbit568 1:4,000) at 4 °C for 48 h. The excess of secondary antibodies was removed by six, 10 min washes in 1xPBS. Sections were then mounted on slides and coverslipped with Aqua PolyMount (PolyMount Sciences, Inc.) and kept at 4 °C.

### Analysis of spine density and mitochondrial morphology

Dendritic spine and mitochondrial morphology quantification was carried out by first identifying 2° or 3° dendritic segments in the basal, apical oblique, or apical tuft dendritic compartments using the cell fill channel only, blinding the experimenter to mitochondrial morphology and density, removing potential bias during segment selection. Individual dendritic segments were then measured in their length manually using Fiji (ImageJ-NIH) or NIS-Elements software (Nikon), with each individual mitochondria length in each dendritic segment subsequently measured manually. The same was done for dendritic spines in a select number of segments. In order to calculate mitochondrial occupancy, the sum of an individual dendritic segment’s mitochondria is divided by the total length of the dendritic segment itself. Spine density similarly is calculated by dividing the total number of dendritic spines by the total length of the dendritic segment.

### Plasmids

The following plasmids were used of generated: pCAG:Cre (Addgene: Cat#13775), pEF1α:flex-tdTomato (Addgene: Cat#28306), pCAG:mito-YFP (Addgene; Cat# 168508)^61^, pCAG:mito-dsRed^62^, pCAG:tdTomato^61^, pCAG:mGreenLantern (Addgene: Cat#164469), pCAG:mtYFP-P2A-tdTomato^61^, pCAG:Kir2.1-T2A-tdTomato was a gift from Massimo Scanziani (Addgene plasmid #60598), pLKO.1:shMTFR1L (GAGTGGAGTGTATCTGCTTAAGGGG)^25^, pCAG:MTFR1LWT^25^, pCAG:MTFR1LS103D/S238D^25^, pLKO.1:shMFF (CCGGGATCGTGGTTACAGGAAATAA – TRCN0000174665)^7^, pLKO.1:shOPA1 (CCGACACAAAGGAAACTATTT – TRCN0000091111) and pLKO.1:shNT (CGTTAATCGCGTATAATACGCGTAT)^25^. pCMV6 Lphn3 (MR212027), pGFP-C-shLenti scrambled negative control (TR30021) and pGFP-C-shLenti Lphn3 shRNA-D (GTATGTTGGCTTCGCCTTGACACCTACTT, custom) were purchased from Origene. Wild-type (active) and mutant (inactive) Kir2.1:T2A:dTomato sequences were PCR amplified from plasmids (Addgene plasmids #60598 and #60644) and subcloned into the pAAV-hSyn-DIO-mCherry backbone (Addgene plasmid #50459) in replacement of mCherry using AscI and NheI restriction. pCAG mt-paGFP:p2a:mt-mScarlet was created by cloning a DNA gene block (IDT) encoding 2xmt-paGFP (2 Cox8 matrix targeting sequences followed by the DNA encoding photoactivatable GFP), a self-cleaving peptide sequence (p2a), and a 2xmtmScarlet (2 Cox8 matrix targeting sequences followed by the DNA encoding mScarlet) into the pCAG backbone via restriction digest with Xho1 and Not1 to enable equal expression and robust localization of the two reporters to the mitochondrial matrix. pCAG-NES (nuclear export sequence)-jRGECO1a was generated by subcloning a NES-jRGECO1a insert (from pAAV-Syn-NES-jRGECO1a-WPRE, Addgene #100854) into a pCAG backbone.

### In vivo two-photon calcium imaging

We used a 2-photon imaging system described previously^63^. All imaging was conducted using a custom-built 2-photon 8 kHz resonant scanner using large aperture fluorescence collection optics (primary dichroic: 45.0 ×65.0 ×3.0 mm T865lpxrxt Chroma, US; secondary dichroic: 52.0 ×72.0 ×3.0 mm FF560-FDi02-t3 Semrock, US; green emission filter: 50.8 mm FF01-520/70 Semrock, US; red emission filter: 50.8 mm FF01-650/150 Semrock, US; GaAsP PMTs for green and red channels, PMT2101 Thorlabs, US) paired with a Nikon 16x water immersion, 0.8 NA, 3.0 mm working distance objective. Laser power was controlled with a Pockel cell (350-80LA modulator, 320RM 401 driver, Conoptics, US). Image acquisition was controlled through commercial software (ScanImage, Vidriotech, US). jRGECO1a expressing cells were imaged at 1020 nm (Chameleon Ultra II, Coherent, US). All frame scans lasted 1–2 minutes and were acquired at 120-240 Hz (40–60x optical zoom; 64–128 lines/frame, 64–128 pixels/line) from dendrites (oblique and tuft). Dendritic jRGECO1a signals were deconvolved to detect putative events by applying the OASIS software package^64,65^ using an AR1 model with a pre-computed signal decay constant of 300 ms (Arg = 0.97; event threshold = 0.3). All dendritic calcium analyses were performed on the deconvolved events.

Reagent table can be found as Supplementary Table 1.

### Statistics

All statistical analyses were performed in Prism 10 (GraphPad Software). Statistical details including statistical tests, number of brains, dendritic branches, and mitochondria, mean ± deviation and p values are all listed in figures and figure legends.

### Reporting summary

Further information on research design is available in the Nature Portfolio Reporting Summary linked to this article.

### Supplementary information

Supplementary Information

Peer Review File

Reporting Summary

### Source data

Source Data

## Data Availability

The raw imaging data will be provided by the corresponding author upon request. The source data generated in this study are provided in the Source Data file. Source data are provided with this paper.
