# Supplementary material for: Activity-dependent compartmentalization of dendritic mitochondria morphology through local regulation of fusion-fission balance in neurons in vivo
Source: Nat Commun. 2024 Mar 8;15:2142. doi: 10.1038/s41467-024-46463-w (PMC10923867; doi:10.1038/s41467-024-46463-w)
Supplement: Supplementary file 1 — Supplementary Information [file 41467_2024_46463_MOESM1_ESM.pdf]

Supplementary Information

Activity-dependent compartmentalization of dendritic mitochondria morphology through local regulation of fusion-fission balance in neurons *in vivo*

Virga, Hamilton et al.

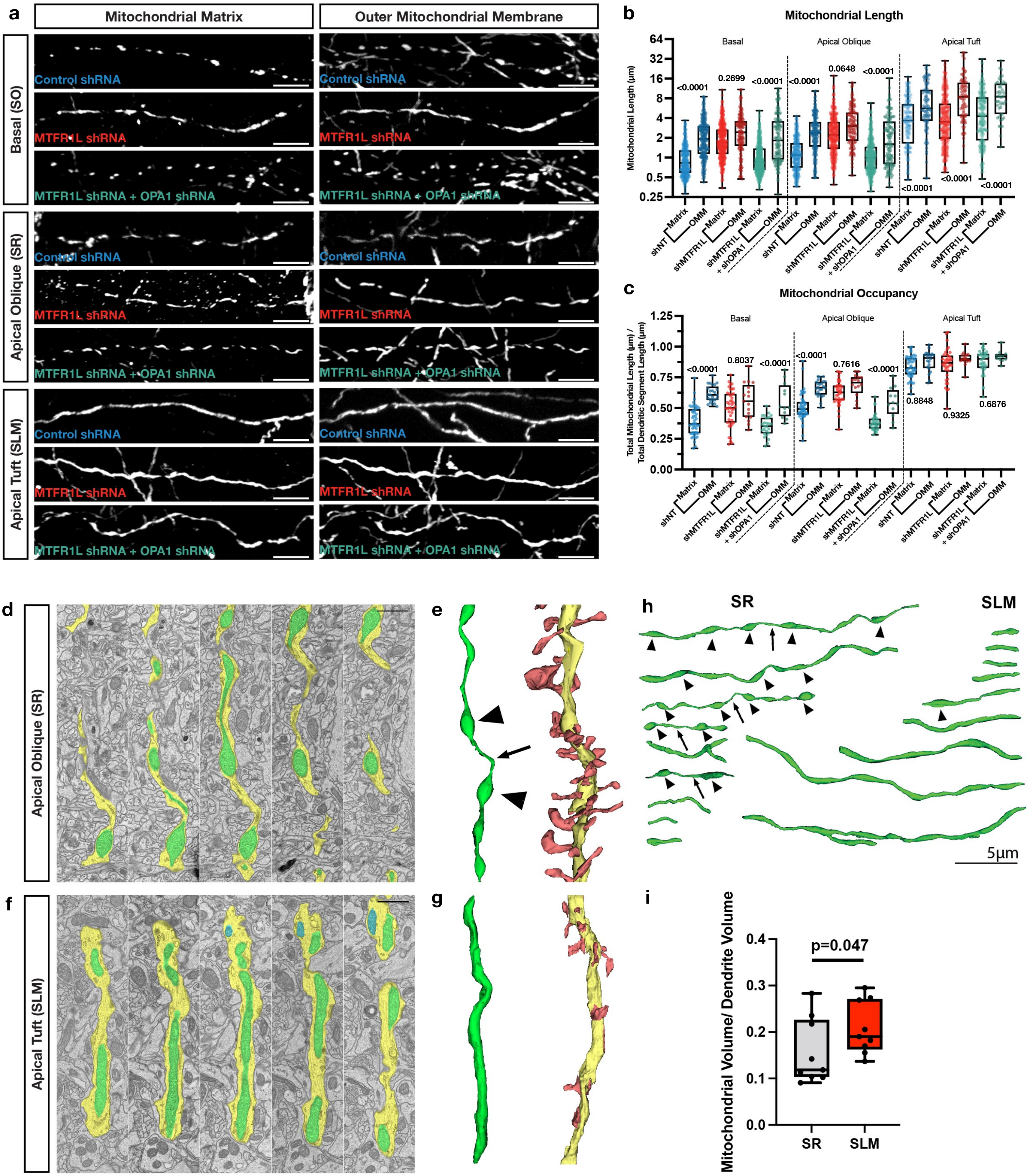
a
Mitochondrial Matrix
Outer Mitochondrial Membrane
Basal (SO)
Apical Oblique (SR)
Apical Tuft (SLM)
Control shRNA
MTFR1L shRNA
MTFR1L shRNA + OPA1 shRNA
b
Mitochondrial Length
Basal
Apical Oblique
Apical Tuft
Mitochondrial Length (μm)
<0.0001
0.2699
<0.0001
<0.0001
0.0648
<0.0001
<0.0001
<0.0001
<0.0001
Matrix
OMM
shNT
shMTFR1L
shMTFR1L + shOPA1
c
Mitochondrial Occupancy
Total Mitochondrial Length (μm) / Total Dendritic Segment Length (μm)
<0.0001
0.8037
<0.0001
<0.0001
0.7616
<0.0001
0.8848
0.9325
0.6876
d
Apical Oblique (SR)
e
f
Apical Tuft (SLM)
g
h
SR
SLM
5μm
i
Mitochondrial Volume/ Dendrite Volume
p=0.047
SR
SLM

Supplemental Figure 1: Compartmentalized mitochondrial morphology visualized using fluorescent OMM and matrix markers as well as 3D serial electron microscopy (3D-SEM). (**a-c**) High magnification representative images of mitochondrial morphology within isolated secondary or tertiary hippocampal CA1 basal, apical oblique, and apical tuft dendrites in which a mitochondrial matrix-targeted fluorescent protein (mt-mTAGBFP2 or mt-YFP), an outer mitochondrial matrix (OMM)-targeted fluorescent protein (mt-ActAmCherry-HA), and cell fill (mGreenLantern or mTAGBFP2—not pictured) were *in utero* electroporated along with either a control shRNA (shNT), MTFR1L shRNA (shMTFR1L), or MTFR1L and OPA1 shRNA (shMTFR1L + shOPA1) (**a**). Quantification of mitochondrial length and occupancy in the basal, apical oblique, and apical tuft dendritic compartments reveals a significant increase in mitochondrial matrix length (**b**) and occupancy (**c**) in basal and apical oblique dendrites when knocking down MTFR1L, which is rescued when also knocking down OPA1 (**Fig. S5e-f**). Note the significant increase in OMM length (**b**) and occupancy (**c**) in all conditions in all dendritic compartments when comparing against mitochondrial matrix length and occupancy. Nevertheless, knocking down shMTFR1L continues to moderately, but significantly, increase the length (**b**) and occupancy (**c**) of the OMM when knocking down MTFR1L, which is rescued when simultaneously knocking down OPA1. There are no significant differences between shNT, shMTFR1L, or shMTFR1L + OPA1 matrix lengths (**b**) or occupancies (**c**), nor between OMM lengths or occupancies. $shNT_{matrix\text{-}basal}$ = 372 mitochondria from 48 dendritic segments; $shNT_{OMM\text{-}basal}$ = 248 mitochondria from 25 dendritic segments; $shMTFR1L_{matrix\text{-}basal}$ = 488 mitochondria from 43 dendritic segments; $shMTFR1L_{OMM\text{-}basal}$ = 145 mitochondria from 17 dendritic segments; $shMTFR1L{+}shOPA1_{matrix\text{-}basal}$ = 515 mitochondria from 25 dendritic segments; $shMTFR1L{+}shOPA1_{OMM\text{-}basal}$ = 122 mitochondria from 12 dendritic segments. $shNT_{matrix\text{-}AO}$ = 273 mitochondria from 46 dendritic segments; $shNT_{OMM\text{-}AO}$ = 188 mitochondria from 24 dendritic segments; $shMTFR1L_{matrix\text{-}AO}$ = 367 mitochondria from 38 dendritic segments; $shMTFR1L_{OMM\text{-}AO}$ = 119 mitochondria from 16 dendritic segments; $shMTFR1L{+}shOPA1_{matrix\text{-}AO}$ = 514 mitochondria from 32 dendritic segments; $shMTFR1L{+}shOPA1_{OMM\text{-}AO}$ = 125 mitochondria from 12 dendritic segments. $shNT_{matrix\text{-}AT}$ = 143 mitochondria from 45 dendritic segments; $shNT_{OMM\text{-}AT}$ = 90 mitochondria from 20 dendritic segments; $shMTFR1L_{matrix\text{-}AT}$ = 330 mitochondria from 38 dendritic segments; $shMTFR1L_{OMM\text{-}AT}$ = 82 mitochondria from 17 dendritic segments; $shMTFR1L{+}shOPA1_{matrix\text{-}AT}$ = 215 mitochondria from 34 dendritic segments; $shMTFR1L{+}shOPA1_{OMM\text{-}AT}$ = 47 mitochondria from 11 dendritic segments. p values are indicated in the figure following a one way ANOVA with Sidak's multiple comparisons test. Data are shown as individual points on box plots with 25th, 50th and 75th percentiles indicated with whiskers indicating min and max values. Scale bar: 5 μm. (**d-g**) Serial transmission electron

microscopy images from the apical oblique dendrites (SR, panel **d**) and the apical tuft (SLM, panel **f**) where the cytoplasm is annotated in yellow and mitochondria in green. Three dimensional reconstructions of the dendritic segments shown in **d** and **f** are shown in panels **e** and **g** where the mitochondria are highlighted in green (left) and the dendritic shaft in yellow with dendritic spines in red. In panel **e**, arrowheads indicate matrix bulges along the continuous mitochondria and the arrow points to a constriction separating two matrix bulges. (**h**) Multiple examples of 3D reconstructed, individual dendritic mitochondria displaying various morphologies and length from SR (left) and SLM (right). Note the presence of frequent matrix bulges (arrowheads) and thin constrictions (arrows) in mitochondria from SR dendritic segments that are rarely observed in SLM dendritic segments. (**i**) Quantification of mitochondrial occupancy defined by volume of dendritic segments reconstructed occupied divided by volume occupied by mitochondria in SR and SO. * $p=0.047$ according to one-tailed Mann-Whitney non-parametric test ($U=21$). Number of dendritic segments and mitochondria reconstructed: SR, $n=76$ individual mitochondria along 9 dendritic segments. SLM, $n=101$ mitochondria along 9 dendritic segments.

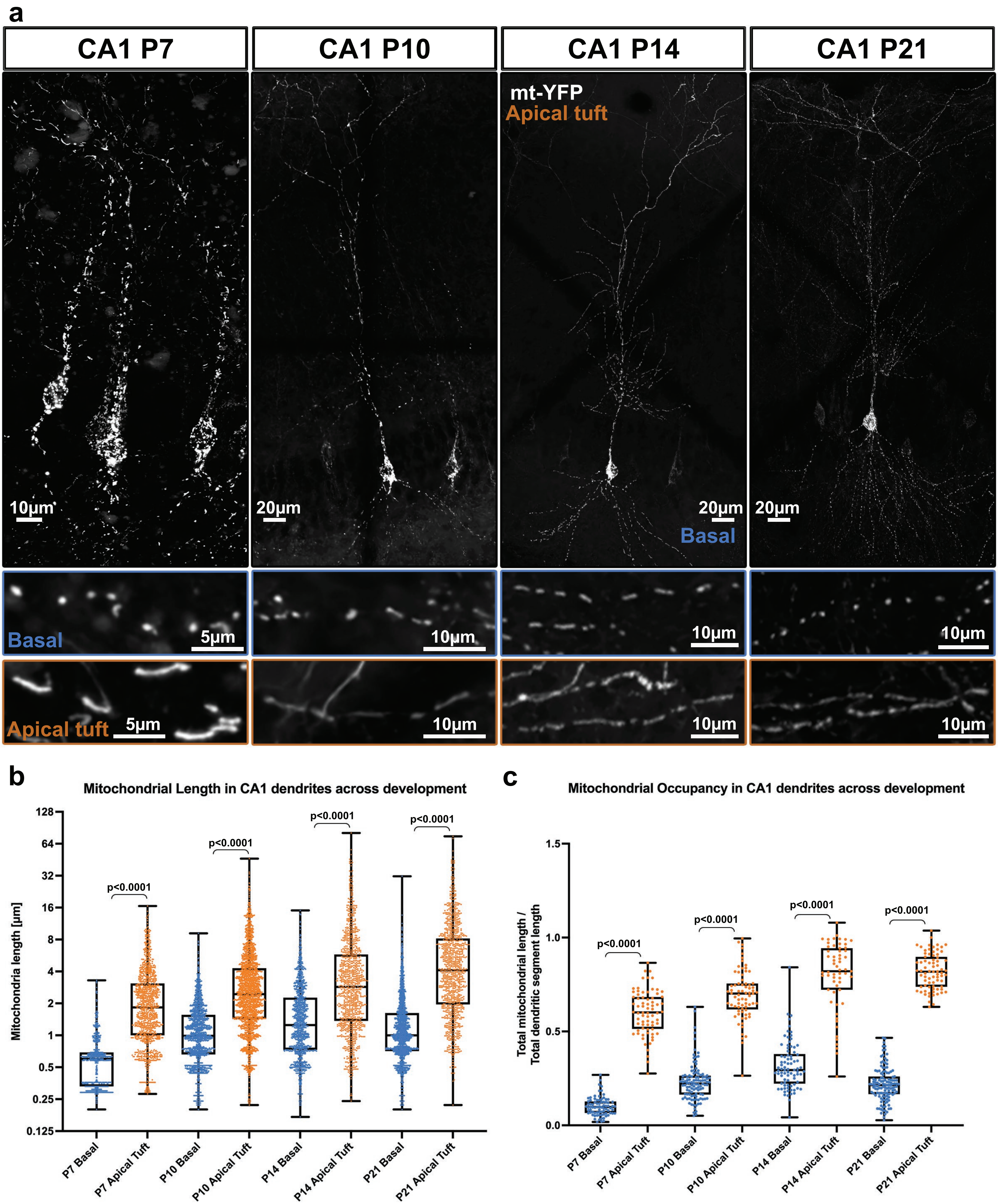
a
CA1 P7
CA1 P10
CA1 P14
CA1 P21
mt-YFP
Apical tuft
10µm
20µm
20µm
20µm
Basal
Basal
5µm
10µm
10µm
10µm
Apical tuft
5µm
10µm
10µm
10µm
b
Mitochondrial Length in CA1 dendrites across development
Mitochondria length [µm]
p<0.0001
p<0.0001
p<0.0001
p<0.0001
P7 Basal
P7 Apical Tuft
P10 Basal
P10 Apical Tuft
P14 Basal
P14 Apical Tuft
P21 Basal
P21 Apical Tuft
c
Mitochondrial Occupancy in CA1 dendrites across development
Total mitochondrial length / Total dendritic segment length
p<0.0001
p<0.0001
p<0.0001
p<0.0001
P7 Basal
P7 Apical Tuft
P10 Basal
P10 Apical Tuft
P14 Basal
P14 Apical Tuft
P21 Basal
P21 Apical Tuft

Supplemental Figure 2: *In vivo* time course of mitochondrial size and occupancy in CA1 pyramidal neurons.

(**a**) Top: Low magnification representative images of mitochondrial morphologies across CA1 pyramidal neurons at the indicated stages of development following IUE of pCAG mtYFP-P2AtdTomato at E15.5. Bottom: Higher magnification boxes of mitochondria morphologies from basal (blue) or apical tuft (orange) dendrites at the developmental stage indicated. (**b-c**) Quantification of mitochondrial length (**b**) and occupancy (**c**) in the basal and apical tufts at the indicated timepoints showing clear, distinct regulation of mitochondrial size and occupancy in the basal dendrites and apical tuft dendrites early in development that is maintained into adulthood. P7basal = 79 segments, 759 mitochondria, mean length = 0.63μm ±0.05μm (SEM), mean occupancy = 10.2% ±0.5% (SEM); P7apical tuft = 72 segments, 1119 mitochondria, mean length = 2.45μm ±0.06μm, mean occupancy = 60.1% ±1.4%; P10basal = 108 segments, 1622 mitochondria, mean length = 1.23μm ±0.02μm, mean occupancy = 23% ±0.9%; P10apical tuft = 73 segments, 2141 mitochondria, mean length = 3.56μm ±0.08μm, mean occupancy = 69.5% ±1.5%; P14basal = 82 segments, 1265 mitochondria, mean length = 1.79μm ±0.05μm, mean occupancy = 31.6% ±1.4%; P14apical tuft = 59 segments, 1022 mitochondria, mean length = 5.1μm ±0.22μm, mean occupancy = 79.6% ±2.4%; P21basal = 127 segments, 2154 mitochondria, mean length = 1.42μm ±0.03μm, mean occupancy = 21.8% ±0.7%; P21apical tuft = 75 segments, 1036 mitochondria mean length = 6.48μm ±0.23μm, mean occupancy = 81.9% ±1.1%. P21 data is the same as the control tdTomato set used in Figure 2. 3 independent brains were used for each condition. p values are displayed within the figure following a Kruskal-Wallis test. Data are shown as individual points on min to max box plots with 25th, 50th and 75th percentiles.

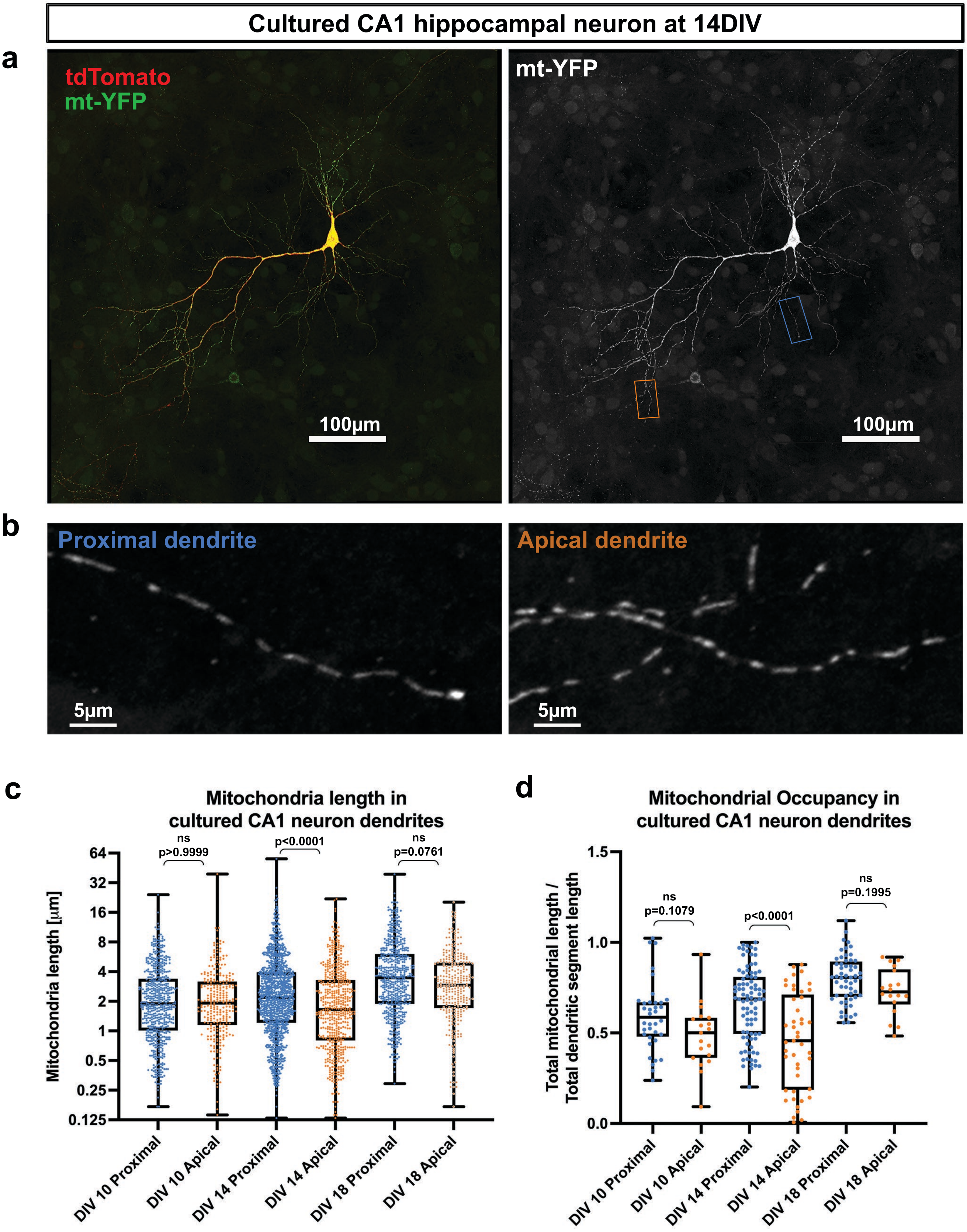

Cultured CA1 hippocampal neuron at 14DIV
a
tdTomato
mt-YFP
100µm
mt-YFP
100µm
b
Proximal dendrite
5µm
Apical dendrite
5µm
c
Mitochondria length in
cultured CA1 neuron dendrites
Mitochondria length [µm]
ns
p>0.9999
p<0.0001
ns
p=0.0761
DIV 10 Proximal
DIV 10 Apical
DIV 14 Proximal
DIV 14 Apical
DIV 18 Proximal
DIV 18 Apical
d
Mitochondrial Occupancy in
cultured CA1 neuron dendrites
Total mitochondrial length /
Total dendritic segment length
ns
p=0.1079
p<0.0001
ns
p=0.1995
DIV 10 Proximal
DIV 10 Apical
DIV 14 Proximal
DIV 14 Apical
DIV 18 Proximal
DIV 18 Apical

Supplemental Figure 3: Dendritic mitochondria morphology observed in CA1 pyramidal neurons *in vivo* are not conserved in cultured CA1 PNs *in vitro*.

(**a**) Low magnification representative images of mitochondrial morphology across a hippocampal neuron in culture following IUE of pCAG mtYFP-P2A-tdTomato at E15.5, followed by culture at E18.5. (**b**) Higher magnification of the indicated boxes in **a**. (**c-d**) Quantification of mitochondrial length (**c**) and occupancy (**d**) in the proximal and apical dendrites at the indicated timepoints showing that in culture the distinct mitochondrial populations observed *in vivo* are absent. 10DIVproximal = 36 segments, 580 mitochondria, mean length = 2.64μm ±0.1μm (SEM), mean occupancy = 59% ±3.2% (SEM); 10DIVapical = 19 segments, 275 mitochondria, mean length = 2.58μm ±0.18μm, mean occupancy = 48.4% ±4.1%; 14DIVproximal = 88 segments, 1199 mitochondria, mean length = 3.2μm ±0.1μm, mean occupancy = 65.7% ±2.1%; 14DIVapical = 45 segments, 527 mitochondria, mean length = 2.56μm ±0.12μm, mean occupancy = 45.5% ±3.9%; 18DIVproximal = 56 segments, 572 mitochondria, mean length = 4.74μm ±0.18μm, mean occupancy = 79.7% ±1.7%; 18 DIVapical = 18 segments, 300 mitochondria mean length = 3.7μm ±0.17μm, mean occupancy = 72.9% ±3%. p values are shown in the figure following a a Kruskal-Wallis test. 3 cultures from independent brains. Data are shown as individual points on min to max box plots with 25th, 50th and 75th percentiles.

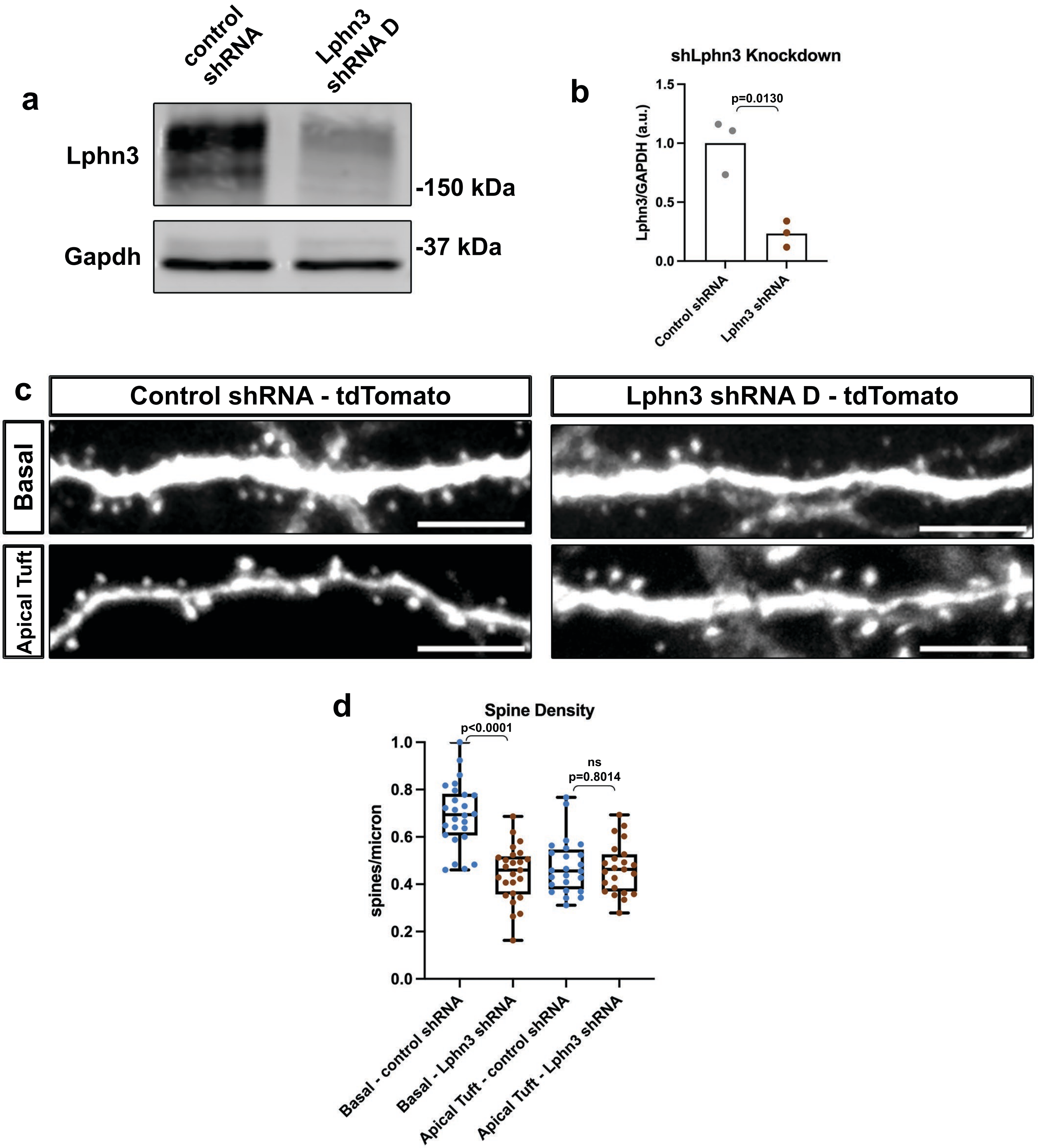
a
control shRNA
Lphn3 shRNA D
Lphn3
Gapdh
-150 kDa
-37 kDa
b
shLphn3 Knockdown
Lphn3/GAPDH (a.u.)
p=0.0130
Control shRNA
Lphn3 shRNA
c
Control shRNA - tdTomato
Lphn3 shRNA D - tdTomato
Basal
Apical Tuft
d
Spine Density
spines/micron
p<0.0001
ns
p=0.8014
Basal - control shRNA
Basal - Lphn3 shRNA
Apical Tuft - control shRNA
Apical Tuft - Lphn3 shRNA

Supplemental Figure 4: Validation of Lphn3 shRNA knockdown construct.

(**a**) Western blot using a mouse Lphn3 antibody (R&D research, top panel) on samples from HEK cells that were transfected with mouse Lphn3 cDNA and either a control or Lphn3 shRNA construct. Gapdh (Cell Signaling Technologies, bottom panel) was used as a loading control (**b**) Relative Lphn3 expression following 72hrs knockdown from 3 independent experiments. Paired t test. p value indicated in the figure. (**c**) High magnification representative images of P21 CA1 dendrite segments electroporated at E15.5 with tdTomato and either control shRNA (left) or Lphn3 shRNA D (right) from basal dendrites in SO (top) or apical tuft dendrites (bottom). (**d**) Quantification of spine density following IUE with control shRNA or Lphn3 shRNA D at P21 demonstrating that Lphn3 knockdown throughout development results in decreased spine density on basal dendrites but not apical tuft dendrites. Control $shRNA_{basal}$ = 26 dendrite segments, mean spine density = 0.69 ±0.03 spines/micron (SEM), Control $_{shRNAapical\ tuft}$ = 24 dendrite segments, mean spine density = 0.48 ±0.02 spines/micron, Lphn3 $shRNA_{basal}$ = 25 dendrite segments, mean spine density = 0.45 ±0.02 spines/micron, Lphn3 $shRNA_{apical\ tuft}$ = 23 dendrite segments, mean spine density = 0.47 ±0.02 spines/micron. 3 independent brains per condition. p values are shown in the figure following a one way ANOVA with Holm-Sidak multiple comparison test. Data are shown as individual points on min to max box plots with 25th, 50th and 75th percentiles. Scale bars are 5 microns.

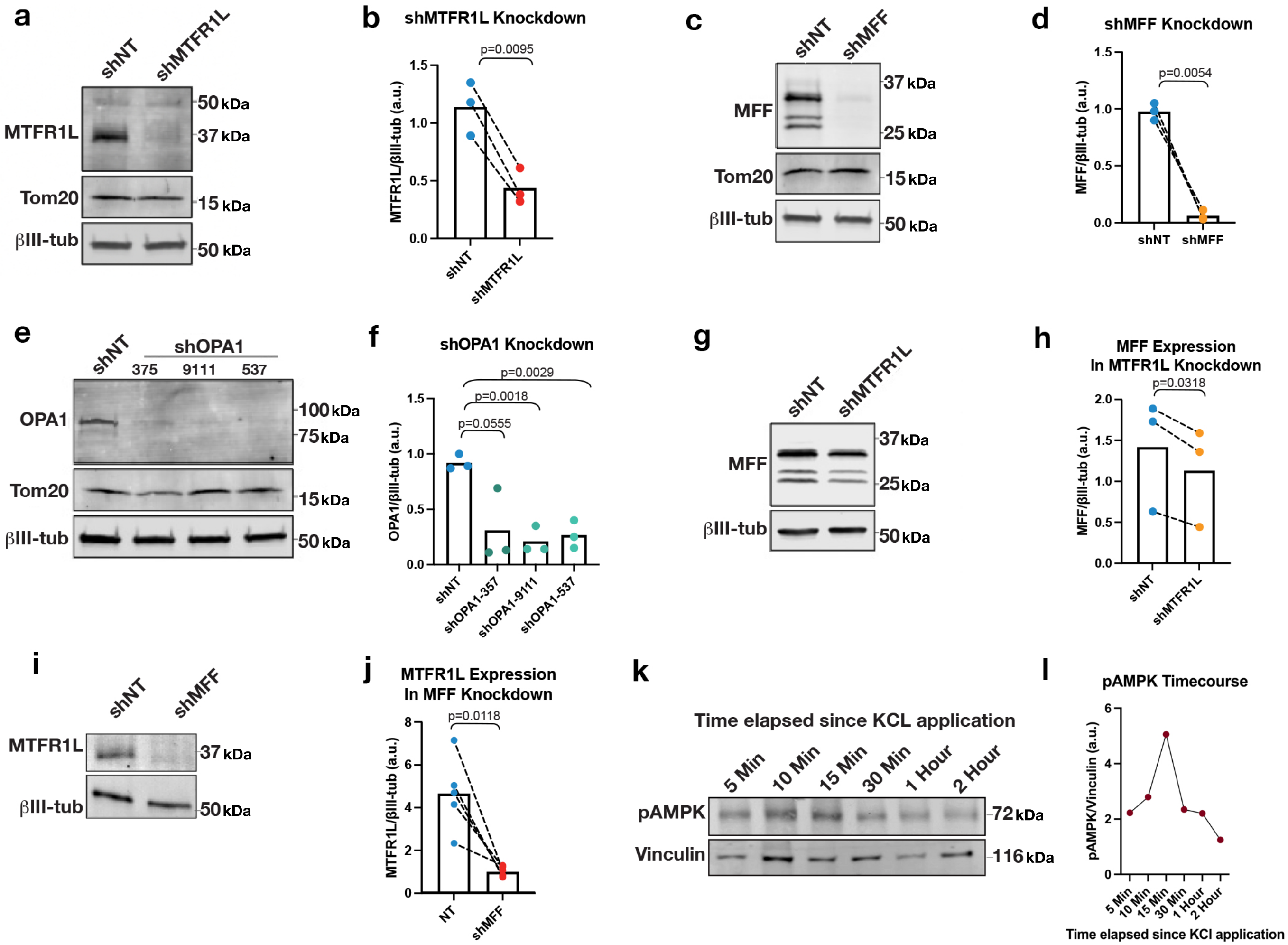

a
shNT
shMTFR1L
MTFR1L
Tom20
βIII-tub
50 kDa
37 kDa
15 kDa
50 kDa
b
shMTFR1L Knockdown
p=0.0095
MTFR1L/βIII-tub (a.u.)
shNT
shMTFR1L
c
shNT
shMFF
MFF
Tom20
βIII-tub
37 kDa
25 kDa
15 kDa
50 kDa
d
shMFF Knockdown
p=0.0054
MFF/βIII-tub (a.u.)
shNT
shMFF
e
shNT
shOPA1
375
9111
537
OPA1
Tom20
βIII-tub
100 kDa
75 kDa
15 kDa
50 kDa
f
shOPA1 Knockdown
p=0.0029
p=0.0018
p=0.0555
OPA1/βIII-tub (a.u.)
shNT
shOPA1-357
shOPA1-9111
shOPA1-537
g
shNT
shMTFR1L
MFF
βIII-tub
37 kDa
25 kDa
50 kDa
h
MFF Expression
In MTFR1L Knockdown
p=0.0318
MFF/βIII-tub (a.u.)
shNT
shMTFR1L
i
shNT
shMFF
MTFR1L
βIII-tub
37 kDa
50 kDa
j
MTFR1L Expression
In MFF Knockdown
p=0.0118
MTFR1L/βIII-tub (a.u.)
NT
shMFF
k
Time elapsed since KCL application
5 Min
10 Min
15 Min
30 Min
1 Hour
2 Hour
pAMPK
Vinculin
72 kDa
116 kDa
l
pAMPK Timecourse
pAMPK/Vinculin (a.u.)
5 Min
10 Min
15 Min
30 Min
1 Hour
2 Hour
Time elapsed since KCl application

Supplemental Figure 5: Validation of MTFR1L, MFF, and OPA1 shRNA constructs.

(**a-b**) Western blot demonstrating shMTFR1L significantly knocks down MTFR1L (37kDa) compared to control shNT in neuronal cultures. β-Tubulin III (βIII-tub, 50kDa) was stained as a control for neuronal content and Tom20 (15kDa) to control for mitochondrial content and quantification (**b**) of shMTFR1L knockdown measuring fluorescence intensity of anti-MTFR1L normalized to anti-βIII-tub. (**c-d**) Western blot demonstrating shMFF significantly knocks down MFF (37-25kDa) compared to control shNT and quantification (**d**) of shMFF knockdown measuring fluorescence intensity of anti-MFF normalized to anti-βIII-tub. (**e-f**) Western blot demonstrating three separate shOPA1s (357, 9111, 537) significantly knocks down OPA1 (78kDa) compared to control shNT and quantification (**f**) of shOPA1 knockdowns measuring fluorescence intensity of anti-OPA1 normalized to anti-βIII-tub. (**g-h**) Western blot probing for MFF in control shRNA and MTFR1L shRNA treated neurons and quantification (**h**) of fluorescence intensity of anti-MFF compared to anti-βIII-tub from **g**. (**i-j**) Western blot probing for MTFR1L in control shRNA and MFF shRNA treated neurons and quantification (**j**) of fluorescence intensity of anti-MTFR1L compared to anti-βIII-tub from **i**. (**k**) Western blot probing for phospho-AMPK in cultures treated with KCl for the indicated timepoint. (**l**) Graph of phospho-AMPK showing that peak activation of AMPK occurs around 15 minutes following KCl induced depolarization. p values are indicated in the figure following paired, two-tailed, t tests. With the exception of k and l (1 experiment), results are from at least 3 independent experiments indicated by individual points on mean bars in each graph.

Supplementary Table 1. Reagents

Fetal Bovine Serum	Gemini Bio-Products	Cat#100-500
B-27 Supplement (50x)	Fisher Scientific	Cat#A3582801
N-2 Supplement (100x)	Thermo Fisher Scientific	Cat#17502048
GlutaMAX™ Supplement	Thermo Fisher Scientific	Cat#35050061
Neurobasal Medium	Thermo Fisher Scientific	Cat#21103049
Hanks' Balance Salt Solution (HBSS), calcium, magnesium, no phenol red	Thermo Fisher Scientific	Cat#14-025-076
Penicillin/Streptomycin	Thermo Fischer Scientific	Cat#15140-122
Papain	Worthington	Cat#LK003178
DNase I	Sigma-Aldrich	Cat#D5025
Poly-D-Lysine	Thermo Fischer Scientific	Cat#A3890401
Fast Green FCF	Sigma-Aldrich	Cat#F7252
32% Paraformaldehyde Aqueous Solution, EM Grade	Electron Microscopy Sciences	Cat#15614-S
Fluoromount-G	Southern Biotech	Cat#0100-01

Glutaraldehyde 25% Aqueous Solution	Electron Microscopy Sciences	Cat#16220
TTX	Hello Bio	Cat#HB1035
Cremophor	Sigma	Cat#238470
AP5	Hello Bio	Cat#HB0225
NBQX	Hello Bio	Cat#HB0442
STO-609	Sigma-Aldrich	Cat#S1318
Compound-991	Selleck Chem	Cat#S8654
DMSO	Santa Cruz Biotechnology	Cat#sc-358801
4-hydroxytamoxifen	TargetMol	Catalog# T4420
DMSO	Sigma	Cat# D2650
PTX	Tocris	Cat# 1128
Mini-Protean TGX (4-20%) SDS-PAGE	BioRad	Cat#4561093
Trans-Blot Turbo Nitrocellulose Transfer Packs, Mini, 0.2 µm	BioRad	Cat#1704158
PVDF Membrane	Immobilon	Cat#IPL0010
Intercept Blocking Buffer	LI-COR Biosciences	Cat#927-60003
N-PER Neuronal Protein Extraction Reagent	Thermo Fisher Scientific	Cat#87792
Phosphotase Inhibitor	Sigma-Aldrich	Cat#P5726
Phosphotase Inhibitor Cocktail 3	Sigma-Aldrich	Cat#P0044
COmplete Protease Inhibitor	Sigma-Aldrich	Cat#11836170001
Benzonase	EMD Millipore	Cat#70664-3
Laemmli Buffer (4x)	BioRad	Cat#1610747
anti-AMPKα (1:2000)	Cell Signaling	Cat#2532S

2-Mercaptoethanol	BioRad	Cat#161-0710
anti-phospho-AMPKα (1:2000)	Cell Signaling	Cat#2535S
anti-MFF (1:1000)	Protein Tech	Cat#17090-1-AP
anti-GFP (1:4000)	Aves Labs	Cat#GFP-1020
anti-DsRED (1:4000)	Takara Bio	Cat#632496
anti-chicken Alexa488 (1:4000)	Invitrogen	Cat#A11039
anti-rabbit Alex568 (1:4000)	Invitrogen	Cat#A11036
anti-MTFR1L (1:2000)	Atlas Antibodies	Cat#HPA027124
anti-phospho-MTFR1L (1:500)	[25]	N/A
anti-Lphn3 (1:1000)	R&D Systems	Cat#MAB5916
anti-Gapdh (1:5000)	Cell Signaling Technologies	Cat#2118
anti-rabbit IRDye800CW (1:10000)	LI-COR	Cat#926-32211
anti-mouse IRDye800CW (1:10000)	LI-COR	Cat#926-32210
anti-rabbit IRDye680RD (1:10000)	LI-COR	Cat#926-368071
Revert 700 Total protein stain	LI-COR	Cat#926-11010
pCAG-TdTomato	[61]	N/A
pCAG-mtDsRed	[62]	N/A
pCAG-mTAGBFP2	[7]	N/A
pCAG-mtYFP	[61]	N/A
pCAG-mtmTAGBFP2	[61]	N/A
pCAG-Kir2.1 t2a TdTomato	Addgene	#60598
pCMV6-Lphn3	Origene	MR212027
pGFP-C-shLenti scrambled negative control	Origene	TR30021
pGFP-C-shLenti Lphn3 shRNA-D	Origene	Custom

hSyn-DIO-mCherry	Addgene	#50459
pCAG-Kir2.1-T2A-TdTomato	Addgene	#60598
pCAG Kir2.1mut-T2A-TdTomato	Addgene	#60644
pCAG mt-paGFP p2a mt-mScarlet	This paper	N/A
FIJI-ImageJ	[65]	https://imagej.nih.gov/ij/
GraphPad PRISM	GraphPad Software Inc.	https://www.graphpad.com/scientific-software/prism/
Trans-Blot Turbo	BioRad	
Vibratome - Leica VT1200	Leica	
Picospritzer III	Parker Hannifin	
ECM 830 Electro Square Porator	BTX	
A1R Confocal Microscope with NISElements	Nikon	https://www.microscope.healthcare.nikon.com/products/confocal-microscopes/a1hd25-a1rhd25
Ti2-E widefield microscope with NISElements	Nikon	https://www.microscope.healthcare.nikon.com/products/inverted-microscopes/eclipse-ti2-series
