# Supplementary material for: Activity-dependent compartmentalization of dendritic mitochondria morphology through local regulation of fusion-fission balance in neurons in vivo
Source: Nat Commun. 2024 Mar 8;15:2142. doi: 10.1038/s41467-024-46463-w (PMC10923867; doi:10.1038/s41467-024-46463-w)
Supplement: Supplementary file 3 — Reporting Summary [file 41467_2024_46463_MOESM3_ESM.pdf]

nature portfolio

Corresponding author(s): Polleux, Franck
Lewis, Tommy

Last updated by author(s): Feb 14, 2024

Reporting Summary

Nature Portfolio wishes to improve the reproducibility of the work that we publish. This form provides structure for consistency and transparency in reporting. For further information on Nature Portfolio policies, see our Editorial Policies and the Editorial Policy Checklist.

Statistics

For all statistical analyses, confirm that the following items are present in the figure legend, table legend, main text, or Methods section.

n/a	Confirmed	
☐	☒	The exact sample size (*n*) for each experimental group/condition, given as a discrete number and unit of measurement
☐	☒	A statement on whether measurements were taken from distinct samples or whether the same sample was measured repeatedly
☐	☒	The statistical test(s) used AND whether they are one- or two-sided<br>*Only common tests should be described solely by name; describe more complex techniques in the Methods section.*
☐	☒	A description of all covariates tested
☐	☒	A description of any assumptions or corrections, such as tests of normality and adjustment for multiple comparisons
☐	☒	A full description of the statistical parameters including central tendency (e.g. means) or other basic estimates (e.g. regression coefficient) AND variation (e.g. standard deviation) or associated estimates of uncertainty (e.g. confidence intervals)
☐	☒	For null hypothesis testing, the test statistic (e.g. *F*, *t*, *r*) with confidence intervals, effect sizes, degrees of freedom and *P* value noted<br>*Give P values as exact values whenever suitable.*
☒	☐	For Bayesian analysis, information on the choice of priors and Markov chain Monte Carlo settings
☒	☐	For hierarchical and complex designs, identification of the appropriate level for tests and full reporting of outcomes
☒	☐	Estimates of effect sizes (e.g. Cohen's *d*, Pearson's *r*), indicating how they were calculated

Our web collection on statistics for biologists contains articles on many of the points above.

Software and code

Policy information about availability of computer code

Data collection	Nikon Elements AR (Build 5.42.03) or Zeiss Black (System 2.3) was used to acquire confocal images. ScanImage (Vidrotech, SI5.7R1) was used for acquire 2-photon images. Licor Image studio (Version 5.4) was used to acquire western images.
Data analysis	Nikon Elements AR (Build 5.42.03) or Fiji (Version 1.54h) was used for analysis of confocal images. Licor Image studio (Version 5.4) was used to analyze western images. Prism 10.1.1 was used to perform statistical analysis. Oasis (0.2.0) was used for analyzing calcium dynamics.

For manuscripts utilizing custom algorithms or software that are central to the research but not yet described in published literature, software must be made available to editors and reviewers. We strongly encourage code deposition in a community repository (e.g. GitHub). See the Nature Portfolio guidelines for submitting code & software for further information.

Data

Policy information about availability of data

All manuscripts must include a data availability statement. This statement should provide the following information, where applicable:

- Accession codes, unique identifiers, or web links for publicly available datasets
- A description of any restrictions on data availability
- For clinical datasets or third party data, please ensure that the statement adheres to our policy

The raw imaging data will be provided by the corresponding author upon request. The source data generated in this study are provided in the Source Data file. Source data are provided with this paper.

Research involving human participants, their data, or biological material

Policy information about studies with human participants or human data. See also policy information about sex, gender (identity/presentation), and sexual orientation and race, ethnicity and racism.

Reporting on sex and gender	N/A
Reporting on race, ethnicity, or other socially relevant groupings	N/A
Population characteristics	N/A
Recruitment	N/A
Ethics oversight	N/A

Note that full information on the approval of the study protocol must also be provided in the manuscript.

Field-specific reporting

Please select the one below that is the best fit for your research. If you are not sure, read the appropriate sections before making your selection.

☒ Life sciences ☐ Behavioural & social sciences ☐ Ecological, evolutionary & environmental sciences

For a reference copy of the document with all sections, see nature.com/documents/nr-reporting-summary-flat.pdf

Life sciences study design

All studies must disclose on these points even when the disclosure is negative.

Sample size	No statistical method was used to predetermine sample sizes. Sample sizes were determined based on obtaining large enough data points per condition to test for normality of distribution and perform appropriate statistical analysis. Sample sizes are similar to or larger than similar publications in the field. Sample sizes are sufficient for our claims based on statistical significance.
Data exclusions	No data was excluded from the analysis
Replication	Each set of results reported in this study were reproduced at least 3 times in independent experiments. Information is included in the figures or legends.
Randomization	Embyros selected for ex utero or in utero electroporation were randomly selected for plasmid injection and primary cultures without regard to sex. Embryos were also randomly assigned to experimental or control group without regard to sex.
Blinding	Investigators were not blind to experimental condition during data collection as either litters of mice from in utero electroporation were either control or experimental, or in the case of being from the same litter one hemisphere denoted control and the other the experimental group. Additionally, the investigator performing the surgical procedure was usually the same as the investigator performing the data collection. Investigators were blind to the experimental conditions during the analysis stage following file renaming paradigms to blind the investigator to condition.

Reporting for specific materials, systems and methods

We require information from authors about some types of materials, experimental systems and methods used in many studies. Here, indicate whether each material, system or method listed is relevant to your study. If you are not sure if a list item applies to your research, read the appropriate section before selecting a response.

Materials & experimental systems

n/a	Involved in the study	
☐	☒	Antibodies
☐	☒	Eukaryotic cell lines
☒	☐	Palaeontology and archaeology
☐	☒	Animals and other organisms
☒	☐	Clinical data
☒	☐	Dual use research of concern
☒	☐	Plants

Methods

n/a	Involved in the study	
☒	☐	ChIP-seq
☒	☐	Flow cytometry
☒	☐	MRI-based neuroimaging

Antibodies

Antibodies used	mouse anti-LPHN3 (R&D Systems MAB5916, 1:1000), rabbit anti-GAPDH (CST 2118, 1:5000), rabbit anti-AMPKα (Cell Signaling, #2532S, 1:2000); rabbit anti-phospho-AMPKα (Cell Signaling, #2535S, 1:2000); rabbit anti-MFF (Proteintech #17090-1-AP, 1:1000), rabbit anti-MTFR1L (Atlas Antibodies #HPA027124, 1:2000); rabbit anti-phospho-MTFR1L (Gift from the Graham Hardie Lab (original source), S103, 1:500; characterized in ref 25); chicken anti-GFP (Aves Lab #GFP-1020, 1:4000); rabbit anti-dsRed (Takara #632496 1:4000); goat anti-chicken Alexa Fluor 488 (Invitrogen #A11039, 1:4000); goat anti-rabbit Alex Fluor 568 (Invitrogen #A11036); goat anti-rabbit IRDye800CW (LICOR #926-32211, 1:10000); goat anti-rabbit IRDye680RD (LICOR #926-68071, 1:10000); goat anti-mouse IRDye800CW (LICOR #926-32210, 1:10000).
Validation	Antibodies were verified by size on western and response to RNAi. Results are provided in the supplemental data.<br>mouse anti-LPHN3: recognizes human, rat and mouse latrophilin 3 (Uniprot ID: Q9HAR2) in western blots (validated from manufacturer).<br>rabbit anti-Gapdh: recognizes human, mouse, rat, monkey, bovine and pig Gapdh (Uniprot ID: P04406) on western blot. Validated from manufacturer.<br>rabbit anti-AMPKa: recognizes human, mouse, rat, hamster and monkey Ampk alpha (Uniprot ID: Q13131, P54646) on western blot. Validated from manufacturer.<br>rabbit anti-pAMPK: recognizes human, mouse, rate, hamster, monkey, donkey and yeast phophorylated AMPK alpha (Uniprot ID: Q13131, P54646) on western blot. Validated from manufacturer.<br>rabbit anti-MFF: recognizes human, mouse, rat MFF (GenBank assession number: BC000797) on western blot. Validated from manufacturer.<br>rabbit anti-MTFR1L: recognizes human, mouse, rat MTFR1L (Uniprot ID Q9H019) on western blot. Validated from manufacturer.<br>rabbit anti-phospho-MTFR1L: recognizes human and mouse MTFR1L (Uniprot ID Q9H019) phosphorylated at S103 on western blot. Validated in Tilokani et al, Sci Adv 2022 (ref 25).<br>chicken anti-GFP: recognizes green fluorescent protein (GFP) and variants in ELISA, Flow, ICC, IHC and WB. Validated for immunocytochemistry (ICC) in Lewis et al, Nat Comm 2018 (ref 7).<br>rabbit anti-DsRED: recognizes DsRed-Express, DsRed-Express2, DsRed-Monomer, mCherry, DsRed2, E2-Crimson, tdTomato, mStrawberry, and mBanana in ICC and WB. Validated for imunocytochemistry (ICC) in Lewis et al, Nat Comm 2018 (ref7).<br>All secondary antibodies are labeled with the host and species recognized. All secondary antibodies are validated from the manufacturer.

Eukaryotic cell lines

Policy information about cell lines and Sex and Gender in Research

Cell line source(s)	Human Embryonic Kidney 293T/17 (HEK293T) cells were purchased from ATCC (CRL-11268)
Authentication	Not authenticated following direct purchase from ATCC.
Mycoplasma contamination	Cell lines were not tested for mycoplasm.
Commonly misidentified lines<br>(See ICLAC register)	No commonly misidentified lines were used in the study.

Animals and other research organisms

Policy information about studies involving animals; ARRIVE guidelines recommended for reporting animal research, and Sex and Gender in Research

Laboratory animals	Timed-pregnant hybrid F1 control females were obtained by mating inbred 129/SvJ females (Charles River) and C57BL/6J males in house. Homozygous double conditional knockout (cKO) lines AMPKα1F/Fα2F/F 43 were provided by Dr Benoit Viollet (INSERM, Institut Cochin- Paris, France) and homozygous Camkk2-/- 44 were kindly obtained from Dr. Talal Chatila (Harvard Medical School, Boston). Both AMPKα1F/Fα2F/F double cKO and Camkk2-/- timed-pregnant females were obtained by mating homozygous males with females of the same genotype. CD-1 IGS mice (Strain Code: 022) were purchased from Charles River Laboratories. All embryos were electroporated at E15.5. Mice were perfused between P21 and P23 in all main figures. Supplemental figures show ages of mouse perfusion or collection.
Wild animals	No wild animals were used in this study.
Reporting on sex	Sex was not considered as a variable for this study. Mice were randomly assigned to groups during electroporation at E15.5 before sex could be determined.
Field-collected samples	No field-collected samples were used in this study.
Ethics oversight	All experiments involving mice were done according to protocols approved by the Institutional Animal Care and Use Committee (IACUC) at Columbia University, Oklahoma Medical Research Foundation (OMRF) and in accordance with National Institutes of Health guidelines. Animal health and welfare were supervised by a designated veterinarian. All mice were maintained in Columbia University or OMRF animal facilities that comply with all appropriate standards of care, including cage conditions, space per animal, temperature, humidity, food,

water, and 12-hour light/dark cycles.

Note that full information on the approval of the study protocol must also be provided in the manuscript.
