# Supplementary material for: Activity-dependent compartmentalization of dendritic mitochondria morphology through local regulation of fusion-fission balance in neurons in vivo
Source: Nat Commun. 2024 Mar 8;15:2142. doi: 10.1038/s41467-024-46463-w (PMC10923867; doi:10.1038/s41467-024-46463-w)
Supplement: Supplementary file 2 — Peer Review File [file 41467_2024_46463_MOESM2_ESM.pdf]

nature portfolio

Peer Review File

Activity-dependent compartmentalization of dendritic mitochondria morphology through local regulation of fusion-fission balance in neurons in vivo

Open Access This file is licensed under a Creative Commons Attribution 4.0 International License, which permits use, sharing, adaptation, distribution and reproduction in any medium or format, as long as you give appropriate credit to the original author(s) and the source, provide a link to the Creative Commons license, and indicate if changes were made. In the cases where the authors are anonymous, such as is the case for the reports of anonymous peer reviewers, author attribution should be to 'Anonymous Referee' followed by a clear attribution to the source work. The images or other third party material in this file are included in the article's Creative Commons license, unless indicated otherwise in a credit line to the material. If material is not included in the article's Creative Commons license and your intended use is not permitted by statutory regulation or exceeds the permitted use, you will need to obtain permission directly from the copyright holder. To view a copy of this license, visit http://creativecommons.org/licenses/by/4.0/.

REVIEWER COMMENTS

Reviewer #1 (Remarks to the Author):

In a previous study (Lee, A. et al. Nat Commun 13, 4444 (2022).), the authors observed that mitochondrial morphology differs in different compartments within the dendritic arbor of CA1 neurons in the in vivo conditions. In the current study, they demonstrated that this compartment-specific morphology of dendritic mitochondria requires activity-dependent, Camkk2- dependent activation of AMPK. They also showed that the mitochondrial morphology is likely regulated by phosphorylation of the pro-fission Drp1 receptor Mff and the anti-fusion, Opa1-inhibiting protein, Mtfr1l. The subcellular compartmentalization of mitochondrial morphology in neurons is an interesting phenomenon that may reflect the specialized requirement of mitochondrial functions in different compartments.The current study is well-performed and the data quality is good. The logic of the manuscript is clear.

My major concern is that mitochondrial morphology regulation is a complex process of balanced fusion and fission machineries. We can mimic or rescue a phenotype by simply tilt the balance of the fusion and fission without changing the original cause of the problem. For example, we could rescue a fusion defect caused by loss of MFN2 by inhibition of the activity of Drp1. Therefore, when we analyze the cause of a mitochondrial morphology phenotype, direct evidence is more convincing than the evidence got by phenotype rescue or mimicking experiments. In this manuscript, it would be more convincing if we could see the activity of Camkk2 or AMPK was indeed compartmentalized in the CA1 neuron dendritic arbor. Alternatively, the phosphorylation of Mff or Mtfr1l was compartmentalized.

In addition, the relationship between Mff and Mtfr1l was curious. Did these two proteins form a complex? How do they regulate the stability of each other?

Reviewer #2 (Remarks to the Author):

Virga et al. report compartment-specific differences in mitochondrial morphology in CA1 pyramidal neurons. They describe elongated mitochondria in the apical tuft and a more fragmented morphology in the apical oblique and basal dendrites. This difference in morphology depends on synaptic activity and the activation of AMPK by Camkk2. AMPK is suggested to regulate mitochondrial fission by phosphorylating Mff and Mtfr1l. The study addresses an interesting and important question and proposes a mechanism how local synaptic activity regulates mitochondrial fission.

Major points:

- The authors propose that mitochondrial fusion dominates in dendrites but is suppressed by synaptic activity. Their analysis is mainly based on a quantification of mitochondrial length and dendritic occupancy. Analyzing the rate of fission and fusion by imaging mitochondrial dynamics (see e.g. Divakaruni et al. (reference 13)) would provide more direct evidence that mitochondrial elongation depends on a suppression of fusion.

- The authors suggest that synaptic activity and cytoplasmic Ca2+ regulate mitochondrial dynamics but do not directly analyze Ca2+ signaling. The idea that the reduced mitochondrial volume creates a positive-feedback loop by reducing buffering capacity is interesting but not tested experimentally.

- Synaptic activity is manipulated indirectly by expressing the potassium channel Kir2.1 or reducing the number of synapses by a knockdown of latrophilin. Both interventions result in a chronic reduction of synaptic activity. A more physiologically relevant approach to acutely manipulate synaptic activity would provide stronger evidence for the proposed model.

Additional points

- It is not apparent what additional information the quantification of occupancy contributes since mitochondrial elongation automatically increases occupancy.

- More information about image processing and analysis should be included. It is not sufficient to state that ImageJ was used.

- Details of the statistical analysis are missing in the methods section.

Reviewer #3 (Remarks to the Author):

This interesting study by Virga et al. reports novel molecular and cellular effectors that enable synaptic activity to shape the compartment-specific morphology of mitochondria in neuronal dendrites. It

combines molecular, cell culture and in vivo approaches to interrogate the determinants of mitochondrial morphology compartmentalization in hippocampal CA1 dendrites. It includes elegant experiments and sound results, and represents an important addition to a crucial emerging field.

I only have the following minor questions:

1. Page 6 ("…(2)suggests that an activity-dependent signaling mechanism promotes the small mitochondria morphology in SO and SR in vivo…") – couldn't there be alternative explanations, such that the specific molecular KD and reduction in dendritic spines affects the process directly? The 'activity-dependency' is not, in my view, demonstrated through the evidence presented at this step.

2. The same could be applied for the Kir2.1 experiment above, in which the state is not only a deprivation of activity but a rather 'anti-physiological' state induced in the neuron. Please, expand on the possibilities so that the rationale for all these experiments includes all possible explanations.

3. Discussion: it would be important to add a few statements in the discussion regarding to what extent the reported findings reflect the impact of tonic vs phasic synaptic activity.

4. How fast is phosphorylation induced in ("Therefore, our data suggests that presynaptic activity and cytoplasmic Ca2+ dynamics in basal and apical oblique dendrites of CA1 PNs drives the Camkk2-dependent activity which triggers high levels of AMPK kinase activity and phosphorylation of the anti-fusion effector Mtfr1l (Fig. 4; 34) and profission Mff (Fig. 5; 12,24)")?

5. Ref 36 in statement (Mitochondria structure and function have been proposed to play multiple roles beyond ATP generation including control of local protein synthesis 38 and the emergence of synaptic and circuit properties underlying normal brain function 36,39-41) does not reflect well 'normal brain function' – it would be important to cite references in which normal brain function has been related to variation in mitochondrial features; e.g., PMID: 33583561; https://doi.org/10.1101/2021.06.02.446767; etc.

6. Fig 7 is very useful; however, the title "loss of function" is too generic and it does not explain well the nature of specific approaches indicated. Something is missing in linking the central panel with the peripheral ones in the same figure.

Manuscript NCOMMS-23-18852
Virga, Hamilton et al.

Response to the reviewer's comments

We thank all reviewers for their enthusiastic assessment of the manuscript, and for their thoughtful suggestions to help us improve our manuscript. We have performed new experiments to address almost all comments raised by all three reviewers and we think these new results further improve our study.

Reviewer #1 (Remarks to the Author):
In a previous study (Lee, A. et al. Nat Commun 13, 4444 (2022).), the authors observed that mitochondrial morphology differs in different compartments within the dendritic arbor of CA1 neurons in the in vivo conditions. In the current study, they demonstrated that this compartment-specific morphology of dendritic mitochondria requires activity-dependent, Camkk2- dependent activation of AMPK. They also showed that the mitochondrial morphology is likely regulated by phosphorylation of the pro-fission Drp1 receptor Mff and the anti-fusion, Opa1-inhibiting protein, Mtfr1l. The subcellular compartmentalization of mitochondrial morphology in neurons is an interesting phenomenon that may reflect the specialized requirement of mitochondrial functions in different compartments. The current study is well-performed and the data quality is good. The logic of the manuscript is clear.

My major concern is that mitochondrial morphology regulation is a complex process of balanced fusion and fission machineries. We can mimic or rescue a phenotype by simply tilt the balance of the fusion and fission without changing the original cause of the problem. For example, we could rescue a fusion defect caused by loss of MFN2 by inhibition of the activity of Drp1. Therefore, when we analyze the cause of a mitochondrial morphology phenotype, direct evidence is more convincing than the evidence got by phenotype rescue or mimicking experiments. In this manuscript, it would be more convincing if we could see the activity of Camkk2 or AMPK was indeed compartmentalized in the CA1 neuron dendritic arbor. Alternatively, the phosphorylation of Mff or Mtfr1l was compartmentalized.

Unfortunately for the suggested experiments, the tools are either unavailable or incompatible with 2-photon imaging at this time. To our knowledge, no genetically encoded tools exist that would allow us to directly assess Camkk2, Mff or Mtfr1l activity/phosphorylation *in vivo* at this time. For AMPK, there is a reported FRET sensor (AMPKAR-EV, Konagaya et al, Cell Reports, 2017), however it is incompatible with 2-photon excitation as a single wavelength of 2-photon excitation is well known to result in the broad activation of a range of fluorescence sensors (for example 920nm excitation results in simultaneous excitation of both YFP and tdTomato) which will prohibit effective measurement of FRET. This property of 2-photon excitation effectively rules out any sensors requiring measurement with two distinct excitation wavelengths but a single emission channel.
However, we would like to point out that the type of rescue experiments we performed are different from the Mfn2 rescue of Drp1 inhibition as suggested by the reviewer in one important account: we have previously demonstrated (Tilokani et al. Sci. Adv. 2022 (ref 25); and confirmed in the present study – see Figure 9) that Mtfr1l is a direct target of AMPK and that in pyramidal neurons, Mtfr1l is phosphorylated by AMPK in a Camkk2-dependent manner (Mtfr1l phosphorylation increased by neuronal depolarization) is blocked by CAMKK2 inhibitor STO609. Therefore, the rescue experiment (presented in Figure 9d-l) where we completely rescue the increased mitochondrial volume induced by Camkk2 genetic deletion in oblique and basal dendrites of CA1 PNs *in vivo* by expressing a phospho-mimetic form of Mtfr1l is demonstrating without ambiguity that Mtfr1l phosphorylation by AMPK is a key effector mediating the compartmentalization of dendritic mitochondria regulated by Camkk2 *in vivo*.
Also, to address the underlying question of whether the observed mitochondrial morphologies are a result of altered fission or fusion dynamics, we have incorporated a new set of experiments (new Figure 5)

where we directly visualized mitochondrial fission and fusion during increased neuronal activity *in vitro* to show that increased activity results in mitochondrial fission that is Camkk2 dependent (as it is blocked by STO609).

In addition, the relationship between Mff and Mtfr1l was curious. Did these two proteins form a complex? How do they regulate the stability of each other?

We agree that the relationship between Mff and Mtfr1l is interesting. The results presented in Supplemental Figure 5 suggest that Mff does potentially regulate the stability of Mtfr1l, but we think this falls outside of the scope of the current manuscript and would instead be better served as an interesting line of future work.

Reviewer #2 (Remarks to the Author):
Virga et al. report compartment-specific differences in mitochondrial morphology in CA1 pyramidal neurons. They describe elongated mitochondria in the apical tuft and a more fragmented morphology in the apical oblique and basal dendrites. This difference in morphology depends on synaptic activity and the activation of AMPK by Camkk2. AMPK is suggested to regulate mitochondrial fission by phosphorylating Mff and Mtfr1l. The study addresses an interesting and important question and proposes a mechanism how local synaptic activity regulates mitochondrial fission.

Major points:
- The authors propose that mitochondrial fusion dominates in dendrites but is suppressed by synaptic activity. Their analysis is mainly based on a quantification of mitochondrial length and dendritic occupancy. Analyzing the rate of fission and fusion by imaging mitochondrial dynamics (see e.g., Divakaruni et al. (reference 13)) would provide more direct evidence that mitochondrial elongation depends on a suppression of fusion.

We have provided new experimental evidence directly observing mitochondrial dynamics (fission and fusion rates; new **Figure 5**) showing that increased neuronal activity results in increased Camkk2-dependent mitochondrial fission without altering mitochondrial fusion. All our results taken together argue that mitochondrial fusion is normally dominant over fission in pyramidal neuron dendrites but synaptic activity directly induces mitochondrial fission leading to shorter mitochondria. This is in agreement with the previous work of both Divakaruni et al (ref 13) and Li et al (ref 14) where chemical LTP or KCl depolarization also resulted in increased mitochondrial fission.

- The authors suggest that synaptic activity and cytoplasmic Ca2+ regulate mitochondrial dynamics but do not directly analyze Ca2+ signaling. The idea that the reduced mitochondrial volume creates a positive-feedback loop by reducing buffering capacity is interesting but not tested experimentally.

We agree that directly observing calcium dynamics *in vivo* is important, despite being challenging (no previous study has ever measured amplitude and frequency of Ca^{2+} transients in the distal apical tufts of CA1 PNs *in vivo*). To address this, we have performed these challenging imaging and analyzed calcium dynamics (amplitude and frequency (inter-event interval) in apical oblique and apical tuft dendrites from CA1 neurons *in vivo* for the first time (new **Figure 2**). Remarkably, as predicted by our model, these new experiments demonstrate that indeed Ca^{2+} dynamics are both more frequent and of an increased amplitude in apical oblique dendrites than in the apical tufts of CA1 PNs *in vivo*, which is in line with our model that the activity of the Camkk2-AMPK pathway would be higher in apical obliques compared to the tufts of CA1 PNs.

- Synaptic activity is manipulated indirectly by expressing the potassium channel Kir2.1 or reducing the number of synapses by a knockdown of latrophilin. Both interventions result in a chronic reduction of

synaptic activity. A more physiologically relevant approach to acutely manipulate synaptic activity would provide stronger evidence for the proposed model.

We agree that it is interesting to ask if the Ca^{2+} and activity-dependent pathway (Camkk2-AMPK) not only operates during development to shape the compartmentalized mitochondrial morphology characterizing CA1 PNs *in vivo* but also whether this pathway acutely regulates the maintenance of this striking degree of compartmentalization in mature CA1 PNs *in vivo*. To address if neuronal activity is required for maintenance of mitochondria morphology over shorter time periods in mature CA1 PNs, we have performed a new set of experiments where Kir2.1 is not expressed until P21 and in an acute manner (less than 48 hours) through the use of inducible tamoxifen-dependent Cre recombinase (ERT2-Cre-ERT2- see **new Figure 4**). These results clearly demonstrate that acutely reducing neuronal activity over a short period of time in mature CA1 PNs neurons *in vivo* results a significant change in mitochondria morphology in apical oblique and basal dendritic compartments to levels indistinguishable from chronic manipulations throughout development. Coupled with the original Kir2.1 and Lphn knockdown (reducing by 50% the number of synapses received specifically by apical oblique and basal dendrites from CA3) experiments, our results argue that neuronal activity levels play an important role during both the development and in adult for the the maintenance of the compartmentalization of dendritic mitochondrial morphology characterizing CA1 PNs *in vivo*.

Additional points:
- It is not apparent what additional information the quantification of occupancy contributes since mitochondrial elongation automatically increases occupancy.

The mitochondrial occupancy is an index we developed to estimate mitochondrial density (i.e., fraction of dendrites occupied by mitochondria). In the experiments performed in this manuscript mitochondrial length does appear to be highly associated with mitochondrial occupancy, but this does not always have to be the case. For instance, in Lee et al (ref 12) loss of Ulk2 (a mitophagy adaptor) resulted in shorter mitochondria but not a reduction in mitochondrial occupancy following treatment with AB42 oligomers, and was able to rescue spine loss similar to Mff knockdown. This difference is likely important in the context of mitochondrial calcium buffering capacity as mitochondrial matrix volume could be the same with either many short mitochondria or one long mitochondria. Thus, we think it is equally important to show mitochondrial occupancy has changed as well as mitochondrial size.

- More information about image processing and analysis should be included. It is not sufficient to state that ImageJ was used.

Thank you for pointing out this oversite. We have corrected it by adding additional details about how ImageJ was used to perform the analysis in the methods section.

- Details of the statistical analysis are missing in the methods section.

We apologize for this mistake. We have added additional details about the statistical methods in the methods section. We have also added all statistical tests and n numbers in each figure legend.

Reviewer #3 (Remarks to the Author):
This interesting study by Virga et al. reports novel molecular and cellular effectors that enable synaptic activity to shape the compartment-specific morphology of mitochondria in neuronal dendrites. It combines molecular, cell culture and in vivo approaches to interrogate the determinants of mitochondrial morphology compartmentalization in hippocampal CA1 dendrites. It includes elegant experiments and

sound results, and represents an important addition to a crucial emerging field. I only have the following minor questions:

1. Page 6 ("…(2)suggests that an activity-dependent signaling mechanism promotes the small mitochondria morphology in SO and SR in vivo…") – couldn't there be alternative explanations, such that the specific molecular KD and reduction in dendritic spines affects the process directly? The 'activity-dependency' is not, in my view, demonstrated through the evidence presented at this step.

2. The same could be applied for the Kir2.1 experiment above, in which the state is not only a deprivation of activity but a rather 'anti-physiological' state induced in the neuron. Please, expand on the possibilities so that the rationale for all these experiments includes all possible explanations.

We have addressed points 1 & 2 with the addition of two new experiments (new Figures 4 & 5). In Figure 4, we acutely expressed Kir2.1 in mature neurons using an inducible Cre *in vivo* which still resulted in elongated mitochondria. This argues that the previous Kir2.1 and Lphn3 KD experiments are not just a lack of development phenotype but are directly related to changes in activity. Second, after inducing increased neuronal activity with picrotoxin (which reduces GABAergic inhibitory tone), we observed rapid increased Camkk2-dependent mitochondrial fission in dendrites *in vitro*. These results strongly support the argument that neuronal activity directly results in increased mitochondrial fission.

3. Discussion: it would be important to add a few statements in the discussion regarding to what extent the reported findings reflect the impact of tonic vs phasic synaptic activity.

We modified our discussion of these results in light of our new added data using acute reduction of neuronal activity using Cre-ERT2-induction of Kir2.1 expression in mature CA1 PNs *in vivo* and our *in vitro* acute increase in neuronal activity using PTX-mediated reduction of inhibition experiments.

4. How fast is phosphorylation induced in ("Therefore, our data suggests that presynaptic activity and cytoplasmic Ca2+ dynamics in basal and apical oblique dendrites of CA1 PNs drives the Camkk2-dependent activity which triggers high levels of AMPK kinase activity and phosphorylation of the anti-fusion effector Mtfr1l (Fig. 4; 34) and profission Mff (Fig. 5; 12,24)")?

We have added additional data in Supplemental Figure 5k-l showing that AMPK activity increases within 10 minutes and peaks 15 minutes post depolarization.

5. Ref 36 in statement (Mitochondria structure and function have been proposed to play multiple roles beyond ATP generation including control of local protein synthesis 38 and the emergence of synaptic and circuit properties underlying normal brain function 36,39-41) does not reflect well 'normal brain function' – it would be important to cite references in which normal brain function has been related to variation in mitochondrial features; e.g., PMID: 33583561; https://doi.org/10.1101/2021.06.02.446767; etc.

We have added the suggested reference and others to better reflect normal brain function.

6. Fig 7 is very useful; however, the title "loss of function" is too generic and it does not explain well the nature of specific approaches indicated. Something is missing in linking the central panel with the peripheral ones in the same figure.

Thank you for pointing this out. We have redesigned the figure to better represent the specific approaches used and to tie it all together.

REVIEWER COMMENTS

Reviewer #1 (Remarks to the Author):

Although the authors cannot fully address my questions due to technical difficulties, they indeed tried their best. I am satisfied with what they presented in the revised manuscript.

Reviewer #2 (Remarks to the Author):

In their revised manuscript, the authors included an analysis of calcium dynamics in the hippocampus in vivo and the rate of mitochondrial fusion and fission in cortical neurons in culture. These results address major points that were raised and strengthen the conclusion that mitochondrial length depends on neuronal activity.

The authors proposed that mitochondria are elongated in apical tuft dendrites because fusion dominates over fission (Fig. 10). Fig. 5 shows that increased neuronal activity after suppression of GABAergic inhibition stimulates mitochondrial fission. However, these results do not support the conclusion that fusion dominates over fission since the rates of fusion and fission are balanced without experimental intervention (DMSO controls in Fig. 5c). The authors should be more cautious in their interpretation and address this point in their discussion.

Additional points:

- The quantification of mitochondrial fusion and fission (p. 8) is based on a assay that was first described in Karbowski et al. (J. Cell Biol. 164 (4), 2004), which should be cited here.

- Mitochondria in Fig. S3c (DIV 14) appear to be significantly shorter than in Fig. 5a and b. Does this reflect differences between hippocampal (Fig. S3c) and cortical neurons (Fig. 5)? Why did the authors use cortical neurons instead of hippocampal neurons for this assay?

- Fig. 5 is labeled a-c and not a-e as described on p. 8.

- The pCAG mt-paGFP:p2a:mt-mScarlet construct should be described in more detail. What are 2xmt-paGFP and 2xmtmScarlet (p. 17)?

Reviewer #3 (Remarks to the Author):

The authors have addressed all my questions satisfactorily. Still, although the new experiments performed to address my first two questions help providing important information, the causal involvement of activity-dependent is not the only conclusion from their data. Transcriptional changes induced by their manipulations, not implying activity, could also be responsible of the reported changes. I strongly advice to include such alternative explanation in the corresponding Discussion section.

REVIEWER COMMENTS
Revision 2_ NCOMMS-23-18852

We thank all reviewers for their enthusiastic assessment of the manuscript, and for their thoughtful suggestions to help us improve our manuscript. Where necessary, we have responded with text edits in the manuscript.

Reviewer #1 (Remarks to the Author):

Although the authors cannot fully address my questions due to technical difficulties, they indeed tried their best. I am satisfied with what they presented in the revised manuscript.

We thank the reviewer for acknowledging our effort in the revision, and supporting the manuscript.

Reviewer #2 (Remarks to the Author):

In their revised manuscript, the authors included an analysis of calcium dynamics in the hippocampus in vivo and the rate of mitochondrial fusion and fission in cortical neurons in culture. These results address major points that were raised and strengthen the conclusion that mitochondrial length depends on neuronal activity.

The authors proposed that mitochondria are elongated in apical tuft dendrites because fusion dominates over fission (Fig. 10). Fig. 5 shows that increased neuronal activity after suppression of GABAergic inhibition stimulates mitochondrial fission. However, these results do not support the conclusion that fusion dominates over fission since the rates of fusion and fission are balanced without experimental intervention (DMSO controls in Fig. 5c). The authors should be more cautious in their interpretation and address this point in their discussion.

While our hypothesis remains that fusion will dominate over fission in SLM dendrites *in vivo*, or when activity is dampened, we have revised our statement to better reflect the data present in the manuscript.

"Our data strongly supports a model where low, activity-dependent, dendritic Ca^{2+} dynamics inhibits the engagement of the mitochondrial fission machinery,…" now starts the paragraph.

We added "In the future, it will be important to determine if the rates of mitochondrial fusion to fission are unbalanced in distinct dendritic compartments of CA1 hippocampal neurons *in vivo* thus allowing for mitochondrial fusion to dominate, or if other mitochondrial processes (e.g. trafficking, biogenesis or mitophagy) might also play a role in the development of mitochondrial morphological compartmentalization in neuronal dendrites" to end the paragraph.

Additional points:
- The quantification of mitochondrial fusion and fission (p. 8) is based on a assay that was first described in Karbowski et al. (J. Cell Biol. 164 (4), 2004), which should be cited here.

We have rectified this oversight and have included this citation in the manuscript.

- Mitochondria in Fig. S3c (DIV 14) appear to be significantly shorter than in Fig. 5a and b. Does this reflect differences between hippocampal (Fig. S3c) and cortical neurons (Fig. 5)? Why did the authors use cortical neurons instead of hippocampal neurons for this assay?

There are potentially two effects leading to the discrepancy in mitochondrial lengths for figure S3c and Figure 5. First, all the imaging experiments done in figure S3 were done on fixed samples while the imaging in Figure 5 was performed using live imaging. As we have previously shown and discuss in the manuscript, fixation causes some level of shrinkage (ref 28, last paragraph of p. 4) thus it is hard to directly compare mitochondrial lengths in fixed versus live samples. Second, as mentioned by the reviewer the data from figure 5 are in cultured cortical neurons, in which we observe increased dendritic mitochondrial length (ref 7). We performed

the experiments for figure 5 in cortical neurons because we thought that it would increase the overall impact of the findings to show that this Ca^{2+}-dependent fission pathway is active in neuronal cell types other than just hippocampal neurons.

- Fig. 5 is labeled a-c and not a-e as described on p. 8.

This typo has been corrected in the manuscript.

- The pCAG mt-paGFP:p2a:mt-mScarlet construct should be described in more detail. What are 2xmt-paGFP and 2xmtmScarlet (p. 17)?

We have added more details describing the construct in the methods. Essentially, we have encoded two repeats of the Cox8 leader sequence enabling mitochondrial matrix targeting in front of the photoactivatable GFP (pa-GFP), as well as in front of the mScarlet. These are separated by a p2a cleavage sequence to allow expression of both cDNAs from the same CAG enhancer/promoter sequence to provide similar protein expression levels and enhanced mitochondrial targeting of each fluorescent reporter to the mitochondrial matrix.

Reviewer #3 (Remarks to the Author):

The authors have addressed all my questions satisfactorily. Still, although the new experiments performed to address my first two questions help providing important information, the causal involvement of activity-dependent is not the only conclusion from their data. Transcriptional changes induced by their manipulations, not implying activity, could also be responsible of the reported changes. I strongly advice to include such alternative explanation in the corresponding Discussion section.

While we provide no evidence for transcriptional changes causing the phenotypes in our manipulations we have added the following statement to the Discussion.

“Activity-dependent gene transcription has been studied extensively for its role in regulating neuronal morphogenesis and connectivity during development and synaptic plasticity in adult circuits (ref PMID: 30359600) but to our knowledge has never been involved in regulating mitochondria (or any other organelle) biogenesis, structure or function in neurons. Interestingly, in developing astrocytes, mGLuR5 signaling controls their maturation through regulation of PGC1a-dependent mitochondria biogenesis (PMID:33852851). Future investigations will determine if, in neurons, the activity-dependent signaling pathway identified here, operating in a spatially-restricted manner in the dendrites of CA1 PNs *in vivo*, also involves transcriptional and/or translational regulation.”

REVIEWERS' COMMENTS

Reviewer #2 (Remarks to the Author):

The authors have addressed all remaining questions.

Reviewer #3 (Remarks to the Author):

The authors have now also addressed my remaining comment adequately. I do not have any further issues.
